# Axonal autophagic vesicle transport in the rat optic nerve in vivo under normal conditions and during acute axonal degeneration

**DOI:** 10.1186/s40478-024-01791-2

**Published:** 2024-05-29

**Authors:** Xiaoyue Luo, Jiong Zhang, Johan Tolö, Sebastian Kügler, Uwe Michel, Mathias Bähr, Jan Christoph Koch

**Affiliations:** Department of Neurology, University Medicine Göttingen, Göttingen, Germany

**Keywords:** Autophagy, Axotomy, Axonal transport, Dynactin, p150Glued, Optic nerve

## Abstract

Neurons pose a particular challenge to degradative processes like autophagy due to their long and thin processes. Autophagic vesicles (AVs) are formed at the tip of the axon and transported back to the soma. This transport is essential since the final degradation of the vesicular content occurs only close to or in the soma. Here, we established an in vivo live-imaging model in the rat optic nerve using viral vector mediated LC3-labeling and two-photon-microscopy to analyze axonal transport of AVs. Under basal conditions in vivo, 50% of the AVs are moving with a majority of 85% being transported in the retrograde direction. Transport velocity is higher in the retrograde than in the anterograde direction. A crush lesion of the optic nerve results in a rapid breakdown of retrograde axonal transport while the anterograde transport stays intact over several hours. Close to the lesion site, the formation of AVs is upregulated within the first 6 h after crush, but the clearance of AVs and the levels of lysosomal markers in the adjacent axon are reduced. Expression of p150Glued, an adaptor protein of dynein, is significantly reduced after crush lesion. In vitro, fusion and colocalization of the lysosomal marker cathepsin D with AVs are reduced after axotomy. Taken together, we present here the first in vivo analysis of axonal AV transport in the mammalian CNS using live-imaging. We find that axotomy leads to severe defects of retrograde motility and a decreased clearance of AVs via the lysosomal system.

**Supplementary Information:**

The online version contains supplementary material available at 10.1186/s40478-024-01791-2.

## Introduction

Autophagy is essential to maintain cellular homeostasis by clearing damaged or aged intracellular proteins and organelles, especially under stress conditions [1]. However, excessively activated or insufficient autophagy can be detrimental and promote cell death [2, 3]. Accordingly, the correct function of autophagy is important for the fate of the cell. This is particularly important for neurons which are postmitotic cells and thus have to maintain integrity over the whole life-time of the organism. The long cellular processes of neurons present a particular challenge for the autophagic system, especially the thin axon which can extend over a length of more than one meter with a diameter of only a few micrometers. It has been shown before that autophagic vesicles (AVs) are mainly formed at the axon tip, engulf materials there and move the long way back to the soma to complete the degradation process after acidification and transformation to a lysosome only when approaching the soma [4]. Thus, the axonal transport of AVs is an essential yet so far insufficiently studied part of neuronal autophagy [5].

The central role of autophagy becomes very obvious after nerve injury. Within the first hours after axotomy, AVs accumulate at the lesion site and contribute to acute axonal degeneration (AAD) [6]. It is so far not known whether this increase in AVs is caused by increased formation or decreased axonal transport and clearance. While it is beneficial for axonal integrity to inhibit autophagy in the acute phase, intact autophagy is essential for neuronal survival on the long term. A better understanding of the exact pathomechanisms with regards to autophagy after axotomy is essential to develop targeted treatments which could also be applied in other more chronic neurodegenerative conditions.

So far, only a few in vitro and no mammalian in vivo studies of axonal AV transport have been published due to the technical difficulties of imaging axonal transport in vivo. We have established here a set-up which allows the live-imaging of axonal transport of AVs in the rat optic nerve in vivo. This is the first report investigating axonal AV transport in the mammalian central nervous system (CNS) in vivo under physiological conditions and following axotomy.

## Material and methods

### LC3 viral vector

In order to visualize autophagic vesicle transport, rat LC3 cDNA was amplified, fused with the cDNA for the basic red fluorescent protein mScarlet, and finally packed into adeno-associated viral vectors of the serotype 6 (AAV-6). Recombinant AAV particles were produced in transiently transfected HEK293 cells and were purified from the cell lysate by iodixanol step gradient ultracentrifugation followed by heparin affinity chromatography on an Äkta FPLC. The peak eluate was dialyzed against PBS overnight and then frozen in single use aliquots at − 80 °C. The purity of viral particles was confirmed to be > 98% by SDS-PAGE, and vector genome titer was assessed by qPCR. Expression of LC3-mScarlet was driven by a hybrid cytomegalovirus (CMV)-chicken beta-actin (CB) promoter and further augmented by a woodchuck hepatitis virus post-transcriptional regulatory element (WPRE). Before the production of AAV vectors, the sequence of all plasmids was confirmed. The resulting AAV vectors are termed AAV.mScarlet-LC3. The plasmid map is shown in the Fig. 5B.

### Animal experiments

Adult female rats provided by Charles River, weighing between 250 and 350 g at about 3 months of age, were employed in the in vivo experiments. All rats were housed in a room with a temperature of 22 ± 2 °C, a 12/12 h light and dark cycle, and free access to regular food and water. All animal experiments were approved by the local animal research council and complied with the legislation for the management and care of laboratory animals in the state of Lower Saxony, Germany.

### Intravitreal injections of AAV

Under deep anaesthesia with 10% ketamine (95 mg/kg) and 2% xylazine (7 mg/kg), rats were intravitreally injected using a Hamilton syringe. AAV vectors were diluted in PBS to a final volume of 5 μl per eye. Injections were carried out 3 weeks before surgical exposure of the optic nerve with the aid of continuous visual guidance to prevent injury to the lens and retina. The following optimized titers of AAV vectors were applied in order to achieve adequate labeling of the optic nerve without significant toxicity: AAV.mScarlet-LC3 at 5.4 × 10^7^ transforming units (TU) per eye.

### Optic nerve exposure and crush lesion

The surgical exposure of the optic nerve was performed as described before [7]. Briefly, deep anaesthesia with 10% ketamine (95 mg/kg) and 2% xylazine (7 mg/kg) was induced. Breathing and heart rate of the animal, peripheral oxygen saturation as well as pain reflexes were monitored continuously during all procedures and the anaesthesia was adjusted accordingly. The head fur of the rat was shaved off with a razor. The rat was then placed on a heating cushion and stably fixed with a custom-made rat positioning system. Afterwards, a midline incision was made with a scalpel in the skin between the two eyes. The animal was then tilted 30° to the right and placed under a stereomicroscope. After removing the lacrimal gland, a part of the orbital bone was drilled out for later optimal positioning of the microscope objective during live imaging. Subsequently, the superior palpebral and rectus muscles were detached from their proximal tendinous origins using coarse forceps. The eye was rotated downward by pulling the muscles mentioned before. A hook was then placed in the mass of muscles to fix the eye bulb. Care was taken to remove the connective tissue above the optic nerve and keep the central retinal artery intact. The optic nerve sheath was separated longitudinally, layer by layer, eventually exposing the optic nerve. To implement the crush lesion, a polyamide surgical suture (Ethicon, 10-0 Ethilon) was firmly closed around the optic nerve for 30 s to induce AAD.

### Two-photon live-imaging of the optic nerve

After exposing the optic nerve surgically, the orbital cavity was washed multiple times with pre-warmed physiological solution (0.9% NaCl) until the solution became transparent. In vivo live imaging was performed using a two-photon microscope (LaVision BioTec TriM Scope). The objective tip was immersed in the physiological solution above the optic nerve. Fluorescence imaging was used to locate the optic nerve, and the labeled axons in the optic nerve were then properly aligned. A minimum of mercury lamp intensity was applied in order to reduce photobleaching in this process. The position of the objective was sufficiently adjusted including tilting 15° to the right side of the rat, allowing an optical axis perpendicular to the labeled axons. Once the microscope alignment was set up, the rat's position was carefully maintained.

The surgical site was repeatedly rinsed with a pre-warmed physiological solution to maintain a physiological environment during real-time imaging. Simultaneously, the animal was subjected to subcutaneous injection of physiological solution at a dose of 1 ml/hour to prevent dehydration.

By using a custom-built two-photon microscope, 200 time-lapsed images of the optic nerve were acquired with ImSpector software. Briefly described, an optical parametric oscillator (chameleon, coherent) was used as the excitation source. The excitation wavelength of the mScarlet was continuously tuned to 1100 nm. Besides, a Ti:Sapphire laser (coherent), set at 800 nm wavelength, acted as pump-laser. The emitted fluorescence signal was collected through a 20 × immersion Zeiss objective and transferred to a photomultiplier tube (PMT) through a dichroic filter. Imaging areas were selected about 500 μm proximal to the crush site. In order to reduce movement artifacts caused by animal breaths, a custom-built breath trigger was used. An accelerometer was placed at the area of maximum respiratory excursion on the upper lateral thorax of the rat to detect respiratory movements. A custom-built software synchronized respiratory movements of the animal with the image acquisition. The trigger delay varied based on the global image stability, with a preference for short delays (~ 0.2 s) during rapid respiratory rates (> 70/min) and long delays (~ 0.4 s) during slow respiratory rates (< 70/min). The duration of each frame was also calibrated according to the respiratory rate, with values ranging between 0.3 and 0.5 s per frame.

### Processing of in vivo live-imaging data

The raw real-time live imaging of axonal transport was further processed for quantification using a custom-written macro set installed in Image J, which included two parts: "In vivo tracking" and "Kymograph".

At first, the live imaging pictures were registered with the respective ImageJ macro to correct for movement artifacts. Rarely, severely shifted images that could not be corrected by registration were removed from the file using the "Create Sub-Stack" macro. Afterwards, the registered live imaging stack was converted to a projection file. A least 10 axons per file were carefully tracked from the proximal part to the distal part of the optic nerve and added as regions of interest (ROIs). By using the "Kymograph" macro, all tracked axons were automatically transformed into Kymographs one by one. Stationary and motile trajectories of LC3 vesicles in each kymograph were separately marked and manually imported into the ROI-manager. After completion of all track assignments in each imaging sequence, the absolute number and velocity of different LC3 vesicles were automatically calculated through the "Kymograph" macro. Here, we classified LC3 vesicles moving less than a net 5 μm within the 3-min time frame (net velocities ≤ 0.028 μm/s) as stationary ones. Additionally, given the appearance of bulb-like structures in axons following axotomy, trajectories with a diameter greater than 2 µm in the kymographs were excluded from the analysis, as they were more likely to represent degenerated bulbs [8]. At last, the final results were displayed as Excel files for further statistical analysis.

### Neuronal cell culture and viral transduction in vitro

Primary cortical neurons were prepared from E18 Wistar rats embryos as described before [9]. In brief, dissected bilateral cerebral cortices were collected and trypsinized at 37 °C for 15 min. Upon stopping the trypsinization activity, the tissues were gently triturated and homogenized. After centrifugation, cell pellets were suspended in neurobasal medium supplemented with 0.5% human whole transferrin, 1% penicillin–streptomycin-neomycin (PSN), 0.25% L-glutamax (200 mM), and 2% B-27. 60,000 cortical neurons were directly added to the somatic compartment of microfluidic chambers through main channels.

In terms of co-culture of cortical neurons and astrocytes, neonatal cortices were isolated from 1-day-old Wistar rat pups, then shredded, homogenized, and finally incubated with astrocyte culture medium, including low-glucose Dulbecco's modified eagle medium (DMEM), 2.5% PS, and 20% fetal calf serum (FCS) in culture flasks according to the previous protocol [10, 11]. After the removal of microglia by shaking, 30,000 astrocytes were seeded on the soma side of the microfluidic chamber the day before primary cortical neuron culture. The next day, 20,000 primary cortical neurons were added into the somatic compartment.

For viral transduction, AAV.mScarlet-LC3 was diluted in pre-warmed cortex medium and then added to the cortical neurons at 3 h after seeding. The titer of 2 × 10^6^ TU per chamber was found to have an optimal transduction efficacy (around 70% of all neurons) with no signs of cellular toxicity and was therefore selected for all in vitro experiments after previous thorough dose-finding experiments.

### Live-imaging of axonal autophagic vesicle transport in vitro

Microfluidic chambers (Xona Microfluidics, USA) were prepared in accordance with the manufacturer’s recommendations and previously published protocols [12]. 60,000 cortical neurons were seeded into the main channel on one side of the microfluidic chamber, named the soma compartment, and then transduced with AAV.mScarlet-LC3. On DIV 7–9, axons grew long enough to cross the microgrooves and enter the opposite side, called the axonal compartment. Axotomy was carried out by a gentle vacuum pump in the axonal compartment. Passing air bubbles in the main channel caused a mechanical lesion of axons close to the exit of the microgrooves, thus inducing axonal degeneration. On DIV 10, real-time microscopy was performed using an inverted fluorescent microscope (Axiovert, Zeiss) equipped with a cell incubation system to maintain 37 °C and 5% CO_2_. Autophagic vesicle transport in axons labeled with the mScarlet-fluorophore was recorded within the microgrooves approximately 250 µm proximal to the lesion site. Imaging was acquired under 40 × magnification for 10 min at a rate of 0.2 frames/second before and at different time points after axotomy. At least 6 chambers were included in each group.

### Quantification of in vitro time-lapse imaging

To evaluate the kinetics of autophagic vesicle transport after axotomy, in vitro live-imaging was processed and analyzed by KymoAnalyzer, an open-source plugin for ImageJ [13]. This tool automatically categorizes particle tracks and systematically computes a series of parameters of motor-driven transport from time-lapse live-imaging sequences.

Briefly described, 2D kymographs were obtained from the video files through the KymoAnalyzer plugin with coordinates in the x-axis as distance (μm) and the y-axis as time (s), respectively. Particle trajectories were manually tracked using the polyline tool. Quantitative analysis was performed to follow each individual track and calculate the net cargo population and net velocities. Particles moving less than a net 5 μm within the 3-min time frame (net velocities ≤ 0.028 μm/s) were defined as bidirectional or stationary ones.

### Treatment with pepstatin A, BODIPY™ FL Conjugate

BODIPY–pepstatin A (Thermo Scientific, P12271) is a probe used to detect active cathepsin D distribution and trafficking in lysosomes in vitro [14]. On DIV 11, co-cultured cortical neurons and astrocytes transduced with the mScarlet-LC3 viral vector were loaded with BODIPY–pepstatin A (1 μM) for 1 h in both soma and axonal compartments of the microfluidic chamber prior to real-time imaging. Afterwards, the medium at both sides was replaced to remove the fluorescent probe. In order to distinguish individual axons and vesicle fusion rather than spatial overlap, 3-min videos were acquired on the axon side before and at different time points after axotomy. For colocalization analysis between active cathepsin D, as indicated by BODIPY-pepstatin A with the EGFP fluorophore and mScarlet-LC3, the overlapped vesicles labeled with yellow signals were counted manually in individual axons and separately divided by the number of active cathepsin D and LC3 vesicles at each time point.

### Overexpression of the mCherry-EGFP-LC3 tandem

Rat cortical neurons were prepared as described above. A total of 20,000 neurons were transfected with the mCherry-EGFP-LC3 tandem plasmid (5 μg, gift from T. Johansen, University of Tromso, Norway) via electroporation using the Amaxa Nucleofector™ II. On DIV 10, neurons were imaged within an environmental chamber at 37 °C using a PerkinElmer UltraView Vox Spinning Disk Confocal system with a Nikon Eclipse Ti inverted microscope. Time-lapse videos were acquired at a frame rate of 1 frame/sec for 3 min before, every two hours until four hours after axotomy. Kymographs were generated by the "Multi Kymograph" plugin from FIJI and the number of punctae labeled with mCherry only and those with a double-labeling (mCherry + EGFP) were counted.

### Treatment of cortical neurons with bafilomycin

Rat primary cortical neurons were prepared and 60,000 neurons were seeded into the soma compartment of microfluidic chambers. Transduction with AAV.mScarlet-LC3 was performed following the protocol mentioned before. On DIV 10, neurons were treated with either DMSO alone or bafilomycin A1 (5 nm, Sigma-Aldrich, B1793) dissolved in DMSO for 30 h to block autophagic flux. Live imaging was acquired under 40 × magnification using an inverted fluorescent microscope (Axiovert, Zeiss) equipped with a cell incubation system for 5 min at a rate of 0.2 frames/second before and at different time points after axotomy.

### Immunohistochemistry

Rats were euthanized by insufflation of CO_2_ and subsequent decapitation 6 h post-ONC. Dissected optic nerves were fixed in 4% paraformaldehyde at 4 ℃ overnight and cryoprotected in 30% sucrose for at least 48 h. Longitudinal frozen sections of the optic nerve with 16 µm thickness were obtained using a cryostat (Leica CM3050 S, Germany). Antigen retrieval was performed in cell conditioning solution (pH 8.5) at 50 ℃ for 4 h. After blocking in Dako diluent reagent for 1 h at room temperature, sections were incubated with the following primary antibodies: anti-p-ATG16L1 (1:50, Abcam, ab195242), anti-cathepsin D (1:50, Santa Cruz Biotechnology, sc377124), anti-p150glued (1:50, BD Biosciences, 610474), anti-STX17 (1:50, Abcam, ab229646) and anti-SMI 32 (1:500, BioLegend, 801701). The corresponding secondary antibodies were used hereafter: Cy3 (1:500, Dianova, 115165006), Alexa Fluor 647 (1:250, Thermo Scientific, A31571) and Star 635p (1:250, Abberior, 1002). Finally, DAPI diluted in PBS was applied to stain cell nucleus for 10 min at room temperature and then the sections were mounted with Mowiol. Micrographs were taken with a Zeiss Axioplan microscope at 40 × of 63 × magnification equipped with an apotome module, thus enabling pseudo-confocal imaging. All photographs were acquired at 0.34 μm or 0.24 µm intervals over a 2 μm distance using a motorized stage and merged by orthogonal projection to generate a single unified view of the tissue. Only p-ATG16L1 puncta within axons labeled by SIM-32 were evaluated. For STX17, cathepsin D and p150Glued quantification, only STX17 puncta within axons labeled by previous intravitreal injection of an EGFP viral vector (AAV1/2-hSyn-EGFP [Genbank ID: HQ416702]) 4 weeks before animal sacrifice were evaluated.

### Western blot

6 h after ONC, optic nerves were dissected and excised in both proximal and distal regions, spanning a distance of 1 mm from the lesion site. The untreated contralateral optic nerve was used as control. The samples were homogenized and centrifuged at 1300 rpm for 10 min in the lysis buffer as described previously [15]. Afterwards, the supernatant of each sample was carefully collected, and protein concentration was measured using the bicinchoninic acid (BCA) assay (Thermo Scientific, 23227). Equal amounts of protein (15 μg) were loaded to 4–15% precast gradient gels (BioRad, 4561086) and then transferred to PVDF membranes (GE Healthcare, 10600021). The following diluted primary antibodies were used: anti-LC3B (1:1000, Sigma-Aldrich, L7543), anti-cathepsin D (1:300, Santa Cruz Biotechnology, sc-377124), anti-SQSTM1/p62(1:3000, Sigma-Aldrich, P0067), anti-Dynein (1:500, Covance, MMS-400P), anti-p150Glued (1:500, BD Biosciences, 610474), anti-kif 5 (1:1000, Sigma-Aldrich, 3500282), and anti-GAPDH (1:10,000, Hytest Ltd, 5G4). Subsequently, the corresponding HRP-coupled secondary antibodies were employed: anti-mouse HRP (Cell Signaling Technology, 7076P2) and anti-rabbit HRP (Cell Signaling Technology, 7074P2). Protein bands were visualized by ECL reagents (Amersham Biosciences, RPN2236). Finally, the intensity of each band was evaluated using EvolutionCapt Pluse or ImageJ software, and the resulting outcomes were standardized relative to GAPDH as a loading control.

### Statistical analysis

Statistical analyses were carried out with the GraphPad Prism 8 software. Specifically, a two-tailed unpaired t-test was employed to compare two groups, whereas the difference among multiple groups was evaluated through one-way ANOVA or one-way repeated measurement (RM) ANOVA, followed by Tukey’s post-hoc tests. If the data did not conform to a normal distribution, the Mann–Whitney test was applied in measurements of two groups, while comparisons of multiple groups were conducted by the Kruskal–Wallis test or the Friedman test, followed by Dunn’s post-hoc tests. Error bars represented means ± standard error of the mean (SEM). Significance was determined when P < 0.05. (*P < 0.05; **P < 0.01; ***P < 0.001; ****P < 0.0001; N.S.: not significant).

## Results

### Axonal autophagic vesicle transport in the rat optic nerve in vivo under basal conditions

**Fig. 1:**
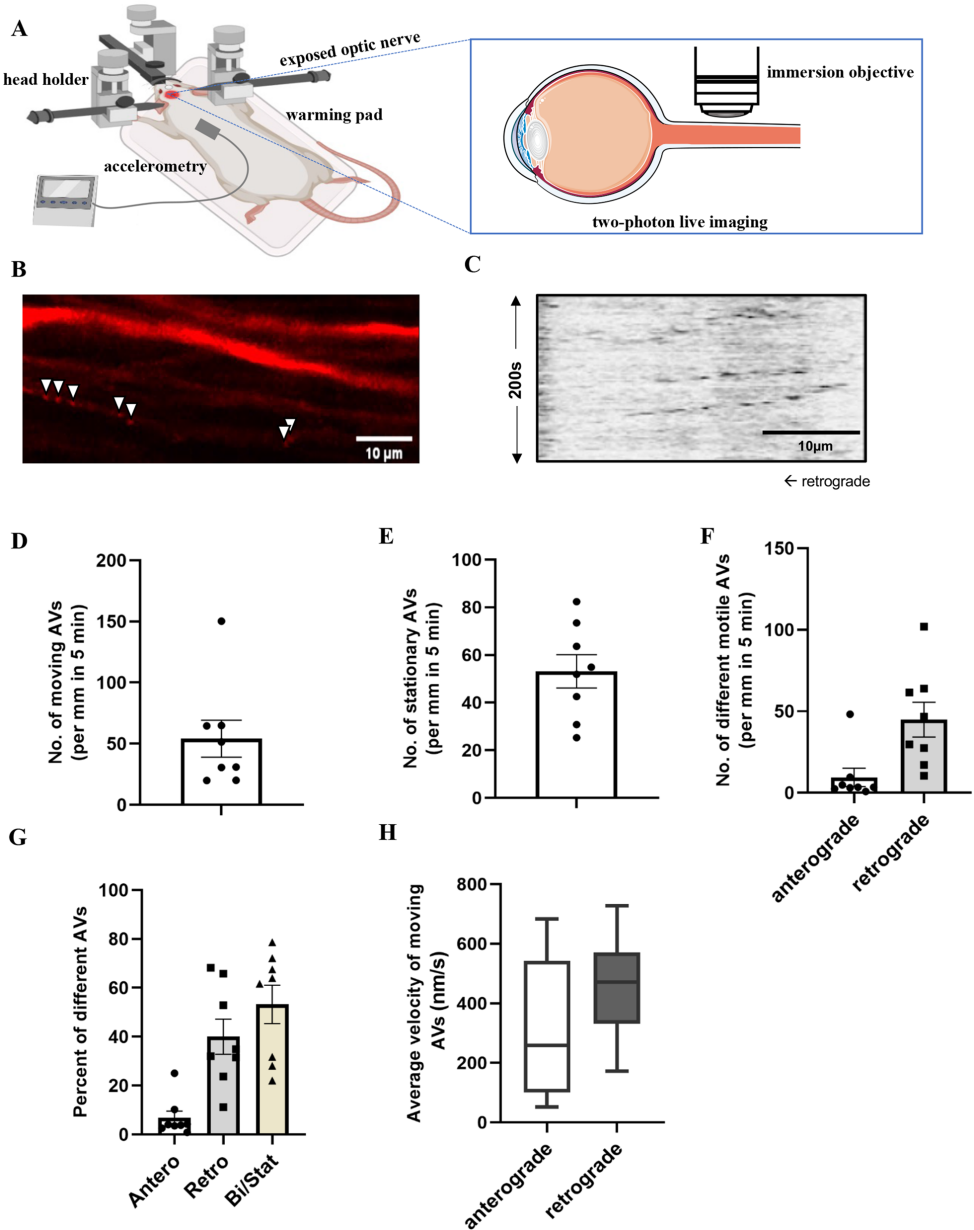
Axonal transport of autophagic vesicles in the optic nerve in vivo. **A** Schematic drawing of the experimental setup for two-photon real-time imaging of the optic nerve. In this experimental setup, the rat is maintained under deep anaesthesia, and its head is securely stabilized using a head holder. Throughout the surgical procedure, the rat is positioned on a heated pad to maintain its body temperature. Vital physiological parameters of the rat are closely monitored throughout the experiment. Using a transorbital approach, the optic nerve is carefully exposed and then subjected to in vivo imaging using a two-photon microscope. **B** Representative two-photon microscopy of the optic nerve. White arrows indicate the LC3 viral vector labeled autophagosomes in the single axon. **C** Representative kymographs of LC3 transport in the rat optic nerve. **D**–**F** Quantification of the number of moving (**D**), stationary (**E**) and moving divided into different transport directions (**F**) LC3 vesicles in the optic nerve of rats under physiological conditions. Error bars represent Mean ± SEM. **G** Quantification of proportions of different LC3 vesicles (anterograde, retrograde, and bidirectional/stationary vesicles) in the physiological situation. Error bars represent Mean ± SEM. **H** Quantification of the average velocity of LC3 vesicles in different directions of movement under physiological conditions. In all quantifications, a minimum of 10 axons per time point per animal was evaluated and a total of 8 animals were included. Figure A modified from biorender, https://app.biorender.com/

Our goal was to analyze axonal AV transport in the mammalian CNS in vivo. We chose the rat optic nerve due to its simple anatomy and good surgical accessibility, which we have already exploited previously [7]. AVs were labeled by intravitreal injections of an adeno-associated viral vector (AAV) expressing the fluorophore mScarlet coupled to LC3 (AAV.mScarlet-LC3). The AAV was injected intravitreally leading to a good transduction of the retinal ganglion cells (RGC) and a strong LC3-signal in the optic nerve axons at 21 days after injection. While there was a significant background signal of the axons representing the cytoplasmatic soluble LC3-I form, we could clearly discriminate labeled vesicles, representing AVs with the membrane-bound LC3-II isoform (Fig. 1B).

The optic nerve was surgically exposed in the orbit behind the eye bulb 21 days after AAV-injection while the animal was under deep anesthesia, as described before [7]. Special care was taken not to damage or stretch the optic nerve and the situs was kept in warmed (37 °C) isotonic solution. The animal was fixed in a special head holder. Image acquisition with a two-photon-microscope was synchronized with the breathing cycle of the animal via accelerometry on the thorax in order to minimize movement artifacts. This set-up (Fig. 1A) allowed for the imaging of axonal AV transport in vivo in adult rats (aged 3 months).

We found that in the imaged region of the optic nerve (1 mm distal from the optic nerve head) there was a density of 107 total AVs per mm over an observation period of 5 min. 53% of those autophagic vesicles were stationary (average 53 ± 7 per mm; Fig. 1D) and 47% were moving (54 ± 2 per mm; Fig. 1E) within 5 min. Among all moving LC3 vesicles, the great majority (85%) was transported retrogradely (Fig. 1F, G). The average velocity of retrograde transport was 461 ± 60 nm/s, which was faster by trend than the average velocity of the anterograde transport with 316 ± 81 nm/s (Fig. 1H).

This strong bias towards retrograde transport of AVs is consistent with previous in vitro studies and our current understanding of axonal autophagy.

### Optic nerve crush disrupts axonal transport of autophagic vesicles

Since autophagy is strongly involved in acute axonal degeneration after axotomy, we next performed live imaging of axonal AV transport during the first six hours after optic nerve crush (ONC). Using a surgical suture, the optic nerve was completely transected. The region 500 μm proximal to the lesion site was imaged with a two-photon-microscope as described above (Video S1).

**Fig. 2:**
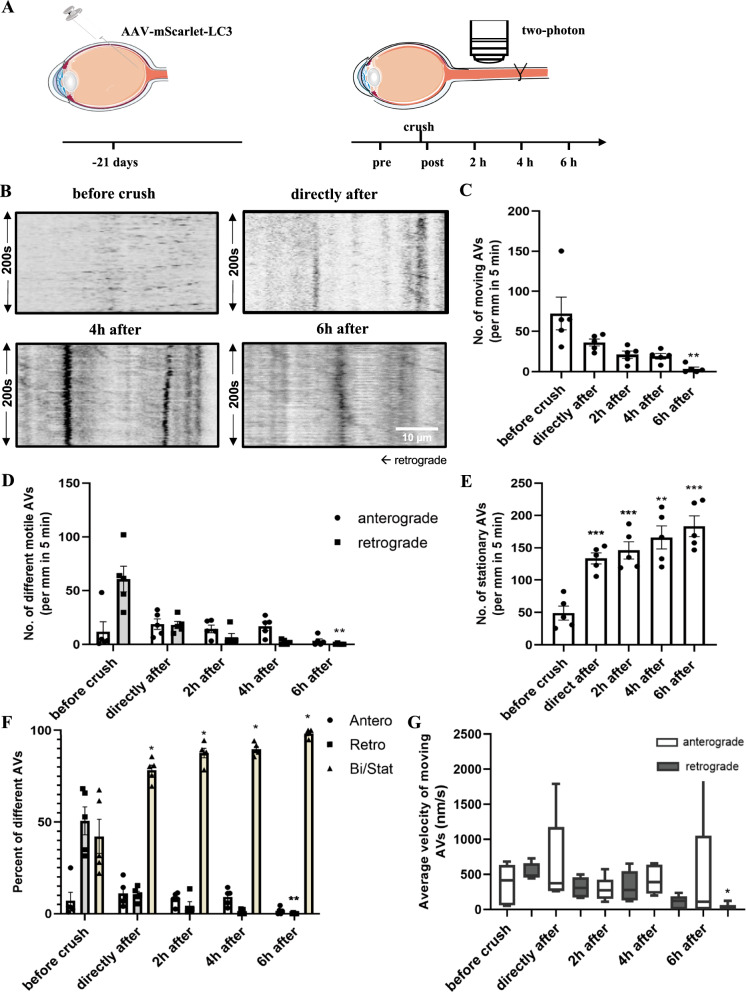
Impact of crush lesion on axonal transport of autophagic vesicles in vivo*.* **A** Schematic diagram of the experimental setup of crush lesion on the optic nerve. The viral vector was intravitreally injected 3 weeks before surgical exposure of the optic nerve. In this model, a suture approximately 1 mm away from the eye bulb was closed for 30 s to create a complete axotomy of the optic nerve. Then the autophagosome transport was evaluated before and over 6 h after crush at 500 μm proximal to the lesion site. **B** Representative kymographs of LC3 transport in the rat optic nerve before and at the given time points after crush lesion. **C**–**E** Quantification of the number of motile and stationary LC3 vesicles in the optic nerve of rats before and at the given time points after crush lesion. Error bars represent Mean ± SEM. **F** Quantification of proportions of different LC3 vesicles (anterograde, retrograde, and bidirectional/stationary vesicles) before and at the given time points after crush lesion. Error bars represent Mean ± SEM. **G** Quantification of the average velocity of different moving LC3 vesicles before and at the given time points after crush lesion. In all quantifications, a minimum of 10 axons per time point per animal was evaluated at approximately 500 μm proximal to the lesion site. A total of 5 animals were included. All statistical analyses were performed by comparing the data at different time points after axotomy to the data before axotomy. *P < 0.05; **P < 0.01; ***P < 0.001; by one-way repeated measures ANOVA and Tukey’s multiple comparisons test or Friedman test and Dunn’s multiple comparisons test based on the normality test of variables

The crush lesion resulted in a rapid breakdown of AV transport, particularly in the retrograde direction (Fig. 2C, D). The number of motile LC3 vesicles decreased by 50% from 72 ± 20 per mm before to 36 ± 4 per mm directly after ONC (Fig. 2C). On the other hand, the number of stationary LC3 vesicles increased 2.7-fold from 49 ± 11 before ONC to 133 ± 9 per mm directly after crush lesion (Fig. 2E, p = 0.0008). These trends were further augmented in the following 6 h with only 3 ± 2 moving LC3 vesicles per mm (p = 0.0014) and 183 ± 16 stationary LC3 vesicles per mm (p = 0.0002) at 6 h after ONC. Interestingly, only the retrograde direction of AV transport was affected with a strong decrease of retrogradely transported vesicles already directly after the crush and further progressing until 6 h after ONC (0.3 ± 0.3 per mm at 6 h after crush compared to 61 ± 12 per mm before crush, representing a 99.5% decrease, p = 0.0032) (Fig. 2D, F). The number of anterograde LC3 vesicles was relatively stable within 4 h after crush injury (before ONC: 12 ± 9 per mm; directly after ONC: 19 ± 5 per mm; 2 h after ONC: 14 ± 4 per mm; 4 h after ONC: 17 ± 4 per mm; 6 h after ONC: 3 ± 2 per mm; no statistically significant difference; Fig. 2D).

In contrast to the strong effects on LC3 vesicle motility, the average velocity of the moving vesicles was relatively stable at all time-points after crush (Fig. 2G). The average velocity of anterograde transport was 360 ± 130 nm/s before crush and did not change significantly during 6 h after ONC. The average velocity of retrograde transport was significantly higher than the anterograde velocity at 540 ± 53 nm/s before ONC and was decreased significantly only at 6 h after axotomy (p = 0.0137).

To confirm that changes in AV trafficking were not caused by the imaging process itself, we imaged rats over 6 h without a lesion. We found no statistically significant changes of all parameters during this imaging time (Video S2 and Fig. S1).

### The formation of autophagosomes is activated after optic nerve crush

During activation of autophagy, the conversion of LC3-I to LC3-II requires the involvement of an LC3 conjugation system comprising ATG16L1/ATG5-12, whereby phosphatidylethanolamine is incorporated into LC3-I. Thus, the ATG16L1-containing complex is indispensable to autophagy. It has been recently demonstrated that the level of phosphorylated ATG16L1 (p-ATG16L1) is directly associated with the autophagy rate [16]. Here, we examined the expression of p-ATG16L1 to determine if autophagy is activated after ONC.

**Fig. 3:**
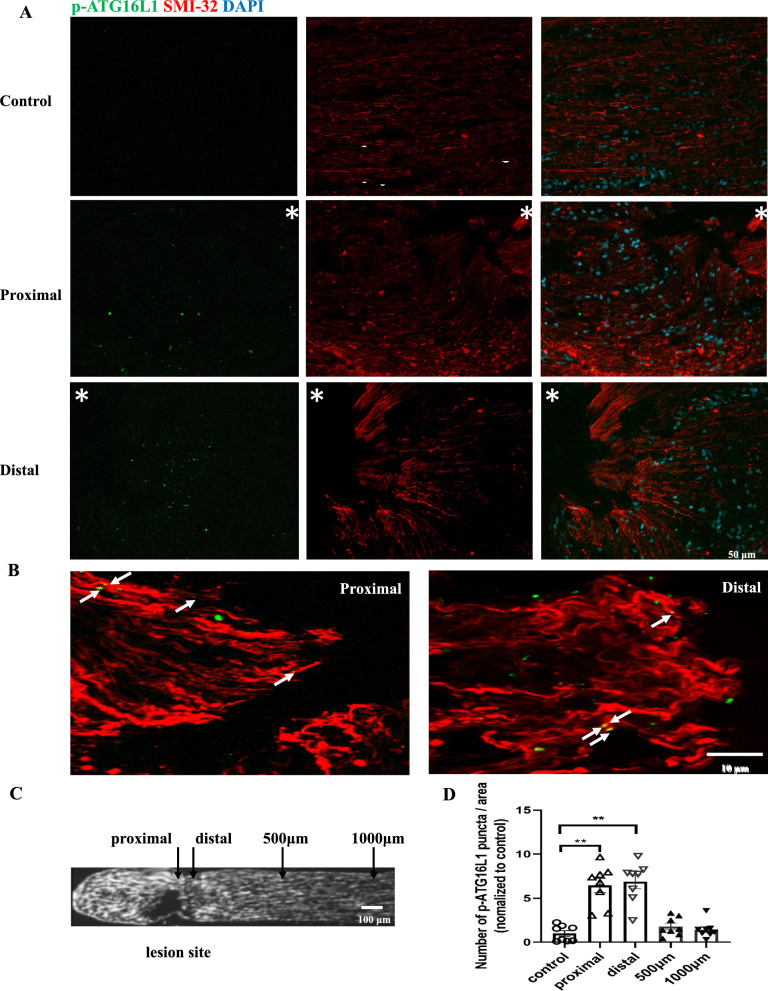
Crush-induced activation of autophagy in the rat optic nerve. **A** Representative double immunofluorescence staining of p-ATG16L1 (green), SMI-32 (red) and DAPI (blue) in the contralateral unlesioned control and different parts of the optic nerve 6 h after crush injury. Asterisks indicate the site of the optic nerve lesion under a 40 × objective. **B** Enlarged views on the two stumps corresponding to the upper panel. Arrows indicate particles of p-ATG16L1 on the SMI-32-labeled axons. **C** Overview image of an optic nerve after crush lesion (labeled with DAPI). **D** Quantitative analysis of the number of axonal p-ATG16L1 puncta in different regions (proximal and distal to the crush site, as well as 500 μm and 1000 μm away from the crush site, as shown in **C**) compared to contralateral uninjured control. The analysis included data from three animals, with four views examined at each region. Data are presented as Mean ± SEM. **P < 0.01, according to Kruskal–Wallis test and Dunn’s multiple comparisons test

At 6 h following ONC, a high number of p-ATG16L1 positive puncta was visible at both proximal and distal terminals of the optic nerve, whereas there was little expression in the untreated contralateral control (Fig. 3A). Further double fluorescence staining showed that some p-ATG16L1-positive puncta overlapped with the axonal marker SMI-32, indicating intra-axonal induction of autophagy (Fig. 3B). The quantification of the number of p-ATG16L1-positive puncta overlapping with SMI-32 showed a significant threefold increase in the regions directly adjacent to the lesion at 6 h after ONC, while there was no change in the regions > 500 µm away from the lesion site (Fig. 3C, proximal part: p = 0.0039; distal part: p = 0.0014).

These data show that autophagy is activated at the tips of lesioned axons within 6 h after lesion.

### Molecular effects of optic nerve crush in vivo

Optic nerve protein lysates were obtained 6 h after the crush lesion from both proximal and distal terminals within 1 mm of the injury site and Western Blot analysis of different proteins was carried out. Protein expression at 6 h after ONC was compared to the unlesioned optic nerve.

**Fig. 4:**
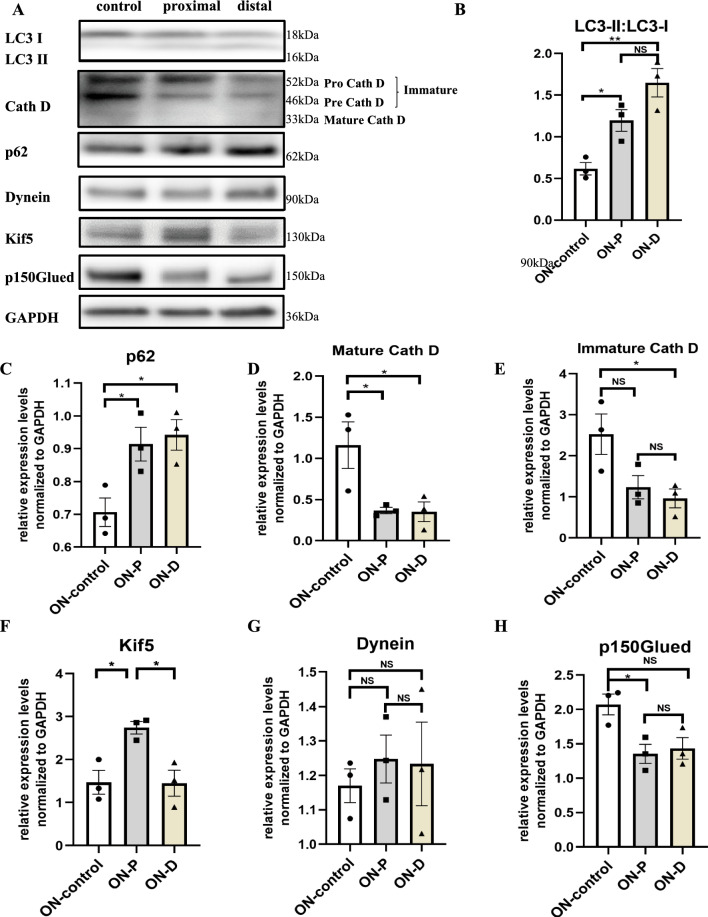
Changes of autophagic pathways and motor proteins in the rat optic nerve 6 h after crush lesion. **A** Representative immunoblots of LC3, p62, Kif5, Dynein, p150Glued, Cathepsin D, and GAPDH 6 h after crush injury in the areas of the optic nerve 1 mm proximal and distal to the lesion site compared with the contralateral side. **B**–**H** Quantifications of the band intensities of LC3 II:LC3 I, p62, Kif5, Dynein, p150Glued, and Cathepsin D normalized to GAPDH as loading control. Error bars represent Mean ± SEM. 3 independent experiments were included. *P < 0.05; **P < 0.01 N.S. no significant difference, according to one-way ANOVA and Tukey multiple comparisons test

The LC3 II:LC3 I ratio, an indicator of autophagic turnover rate [17], was increased on both sides of the optic nerve at 6 h after ONC (Fig. 4A, B, proximal part: p = 0.046; distal part: p = 0.0034). This is consistent with the above p-ATG16L1 staining indicating an enhanced activation of autophagy and an increased formation of autophagic vesicles.

Expression levels of the adaptor protein p62 (SQSTM1), which targets other proteins for selective autophagy, were also elevated significantly at both sides of the crush at 6 h after lesion (Fig. 4C, proximal part: p = 0.0418; distal part: p = 0.0373). This finding could either indicate a reduced autophagic flux or an increased amount of cellular proteins that needs to be degraded or a combination of both.

Next, protein levels of different isoforms of cathepsin D, an essential aspartyl protease that is predominantly located in lysosomes, were evaluated. Cathepsin D is specifically directed to cellular vesicular structures and undergoes a gradual maturation process in the vesicles induced by an acidic environment, thereafter promoting degradation of intravesicular proteins [18]. We found a significant reduction in the levels particularly of the mature isoform of cathepsin D (representing the active lysosomal isoform of cathepsin) on both proximal and distal side of the crush compared to the unlesioned nerve (p = 0.0458 in proximal part and p = 0.0425 in distal part). The immature pre- and pro-cathepsin isoforms were less strongly decreased (p = 0.0468 in the distal part compared to the unlesioned control), indicating that the decrease affects rather activation than translation of cathepsin. This reduction of cathepsin D levels implies a depletion of lysosomal structures around the lesion site. Together with the impaired AV transport in the lesioned axons, it will most likely also be accompanied by a decreased local clearance of autophagic vesicles and reduced autophagic flux.

To further characterize the observed impairment of axonal transport after ONC, we next investigated the levels of relevant motor proteins. Kinesin-1 is a major motor protein required for anterograde autophagic vesicle transport, whereas the dynein-dynactin complex is in charge of retrograde trafficking of AVs [19]. At 6 h after ONC, we found a significant increase of Kif 5, the most abundant member of the kinesin 1 family, in the proximal part of the optic nerve, compared with the distal part (p = 0.0249) and the contralateral unlesioned nerve (p = 0.0268, Fig. 4F). This finding reflects the fact that the anterograde transport on the proximal side was not significantly affected by ONC and thus results in an accumulation of kinesin close to the lesion. On the other hand, no alterations were detected in protein levels of dynein, the major transport protein of retrograde transport. P150Glued is the biggest component of the dynactin complex that is binding to dynein and is essential for the dynein transport function. Its protein levels were significantly reduced after ONC on the proximal side (p = 0.0331) and by trend on the distal side of the lesion (Fig. 4G, H). The reduction of p150Glued could be an explanation for the strong impairment of retrograde transport following ONC.

To confirm that the changes in protein expression found with Western Blotting of optic nerve lysates really apply to RGC axons, we performed an immunohistochemistry against p150Glued and cathepsin D of optic nerves after previous intravitreal injection of AAV overexpressing EGFP to label the axons. We compared optic nerves 6 h after ONC with the unlesioned control and quantified the number of intraaxonal puncta, respectively (Fig. S2). For both, p150Glued and cathepsin D, there was a significant reduction in the number of intraaxonal puncta at 6 h after ONC, in line with our above Western Blot results.

Since these findings imply an impaired fusion of AVs with lysosomes, we also conducted an immunohistochemistry against the vesicle fusion protein Syntaxin 17 (STX17). Interestingly, intraaxonal levels of STX17 were significantly reduced at 6 h after ONC, but only on the proximal side of the lesion (Fig. S2).

### Characteristics of autophagic vesicle transport in vitro differ from in vivo

Given that almost all AV transport studies are currently based on in vitro models, we were curious to investigate potential differences between the in vivo and in vitro settings. Moreover, we wanted to further explore the effects of axotomy on downstream AV transformation which would have been challenging in vivo.

**Fig. 5:**
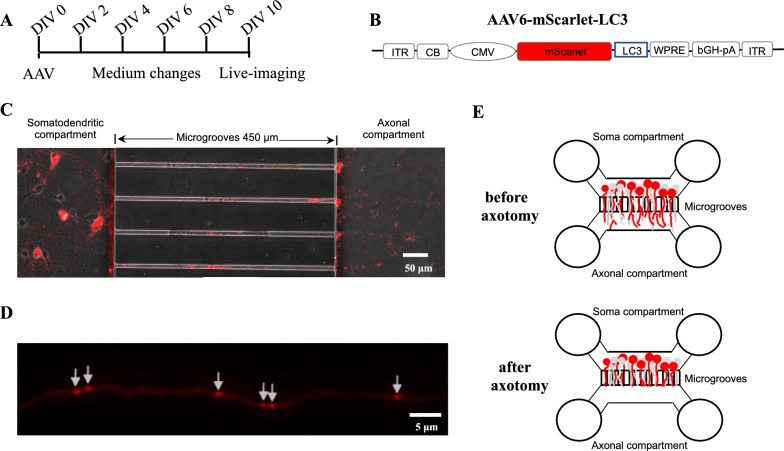
Experimental set-up of an acute axonal degeneration model in vitro. **A** Experimental scheme for the in vitro live imaging procedure. On DIV 0, rat cortical neurons were transduced with the AAV.mScarlet-LC3, which were subsequently imaged on DIV 10. The medium was changed every two days. **B** Vector diagram of AAV.mScarlet-LC3. As an adeno-associated viral vector, it expresses mScarlet fluorophores fused to LC3. ITR: AAV-6 inverted terminal repeat. *CB* chicken beta-actin. *CMV* cytomegalovirus promoter. *WPRE* woodchuck hepatitis virus posttranscriptional regulatory element. *bGH-pA* bovine growth hormone-polyadenylation site. **C** Exemplary image of rat cortical neurons transduced with LC3 AAV vectors in the microfluidic chamber on DIV 10. **D** Representative clearly labeled autophagosomes on the axon transduced with the given LC3 AAV vector on DIV 10. Scale bar: 5 μm. **E** Schematic drawing of axotomy in microfluidic chambers on DIV 10. Each microfluidic chamber comprises 4 wells and 2 compartments connected by 450 μm long microgrooves. The soma compartment serves as the locus for seeding primary cortical neurons. Only axons have the ability to cross the microgrooves and reach the opposite side, named the axonal compartment. Axotomy is performed only in the axonal compartment to induce acute axonal degeneration

To compare the in vivo data with an in vitro set-up using the same AAV in the same species, we cultured rat primary cortical neurons in microfluidic chambers and transduced them on DIV (day in vitro) 0 with the same AAV.mScarlet-LC3 used for the in vivo experiments. Successful transduction was confirmed through detectable mScarlet fluorescence on DIV 10 when mScarlet labeled AVs were easily distinguished (Fig. 5D). To model the crush lesion in vitro, an axotomy model was established based on the description by Park et al. [12], as depicted in Fig. 5E, to explore dynamic alterations of AV transport.

**Fig. 6:**
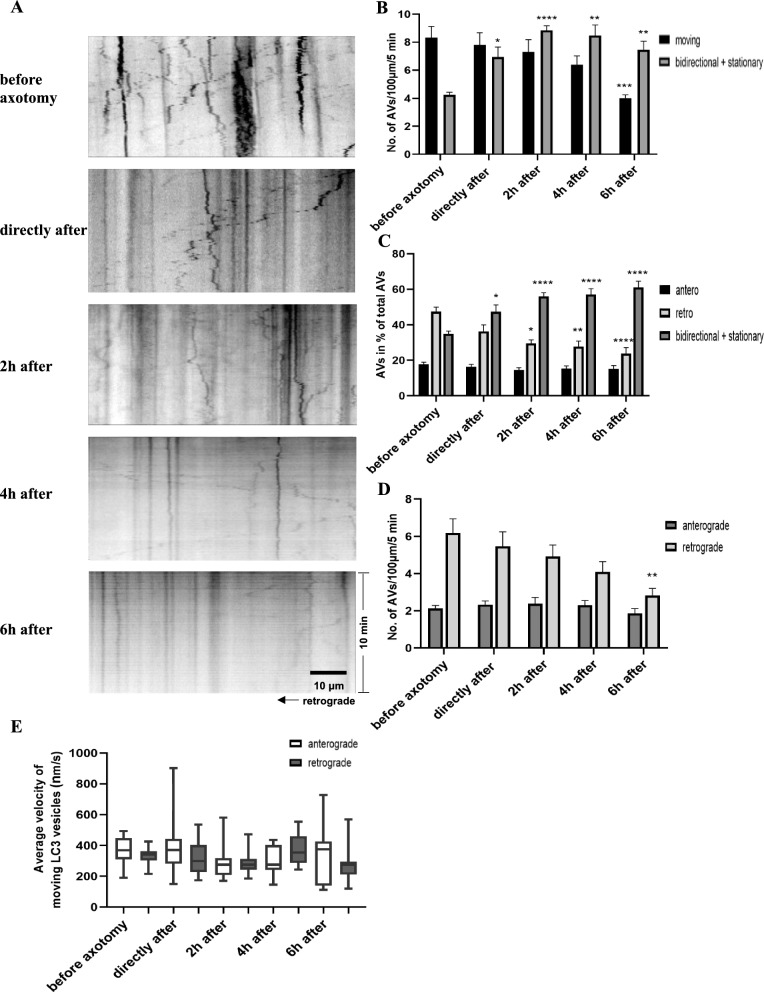
Live imaging of autophagic vesicles in rat primary cortical neurons after axotomy. **A** Exemplary kymographs of autophagosomes along the axons before and at different time points after axotomy within 10 min (x-axis: length of the axon; y-axis: time). **B**–**D** Quantification of autophagosome movements before and after axotomy. Error bars represent Mean ± SEM. **E** Quantification of the average velocity of autophagosomes before and after axotomy. In all quantifications, at least 27 axons in the regions up to 250 μm proximal to the lesion site from 3 independent experiments were included. All statistical analyses were performed by comparing the data at different time points after axotomy to the data before axotomy. *P < 0.05; **P < 0.01; ****P < 0.0001 by one-way ANOVA and Tukey multiple comparisons test or Kruskal–Wallis test and Dunn’s multiple comparisons test according to the normality tests

Similar to our observations in vivo, around 65% of all AVs were moving (8.3 ± 0.8 per 100 μm) while 35% of all AVs were stationary (4.2 ± 0.2 per 100 μm) over an observation period of 5 min (Fig. 6B, C). Among the moving AVs, the majority was transported retrogradely (6.2 ± 0.8 per 100 μm accounting for 73% of all moving vesicles) vs. anterogradely (2.1 ± 0.2 per 100 μm accounting for 27% of all moving vesicles) (Fig. 6C, D), however, the predominance of the retrograde transport was less pronounced compared to in vivo, with the bias towards retrograde transport being 12% less than in vivo. The average velocity of anterograde and retrograde transport was similar (370 ± 0.03 nm/s in anterograde transport and 330 ± 0.02 nm/s in retrograde transport, Fig. 6E), which was 20% slower than the average AV velocity observed in vivo.

Taken together, axonal transport of AVs in primary cortical neurons in vitro as compared to the optic nerve in vivo is characterized by a lower total number of AVs per axon, a higher percentage of moving vesicles and no differences in vesicle transport velocity with regards to transport direction whereas in vivo the retrograde transport was faster than the anterograde transport.

### Axotomy in vitro also impairs the axonal transport of autophagic vesicles

An axotomy was performed by sucking an air bubble through the axonal compartment. Live-imaging was carried out on the axons before, directly after, and every 2 h until 6 h after axotomy within the region extending up to 250 μm proximal from the lesion site (i.e. in the microgrooves) and quantified on kymographs.

After axotomy, there was a rapid increase of the number of stationary AVs from 4.2 ± 0.2 before to 7.0 ± 0.7 per 100 μm directly after axotomy (p = 0.0339). This accumulation of stationary AVs progressed further until 2 h (8.8 ± 0.3 per 100 μm, p < 0.0001). Thereafter the number decreased again until 7.4 ± 0.6 per 100 μm at 6 h after axotomy (p = 0.0041, Fig. 6A, B).

Compared to the in vivo situation, the decrease of all moving AVs was much less pronounced, reaching significance only at 6 h after axotomy (4.0 ± 0.2 per 100 μm, p = 0.0007 Fig. 6D).

With regards to the transport direction, the anterograde transport stayed completely stable over time, while the percentage of vesicles moving retrogradely decreased significantly already at 2 h after axotomy (29.6 ± 1.9%, 4.9 ± 0.6 per 100 μm, p = 0.0141) compared to before axotomy (47.4 ± 2.5%, 6.2 ± 0.8 per 100 μm) and persisted until 6 h after axotomy (23.8 ± 3.4%, 2.8 ± 0.4 per 100 μm, p < 0.0001) (Fig. 6C, D). The velocity of motile AVs was not significantly altered at all observed time points (Fig. 6E).

In comparison to the in vivo situation in the optic nerve, axotomy in vitro thus led to less pronounced but qualitatively similar alterations of axonal AV transport.

### Axotomy leads to decreased fusion of autophagic vesicles with lysosomes

A major finding of the axotomy experiments was that the mobility of AVs is severely impaired after axonal injury. Moreover, cathepsin D levels were reduced significantly in the axon close to the axotomy site in vivo. Since the fusion of AVs with lysosomes during retrograde transport to form mature autolysosomes is an essential step for the degradation of autophagic substrates and thus for the clearance of autophagosomes [4], we wondered if the fusion of AVs and lysosomes is affected after axonal injury.

**Fig. 7:**
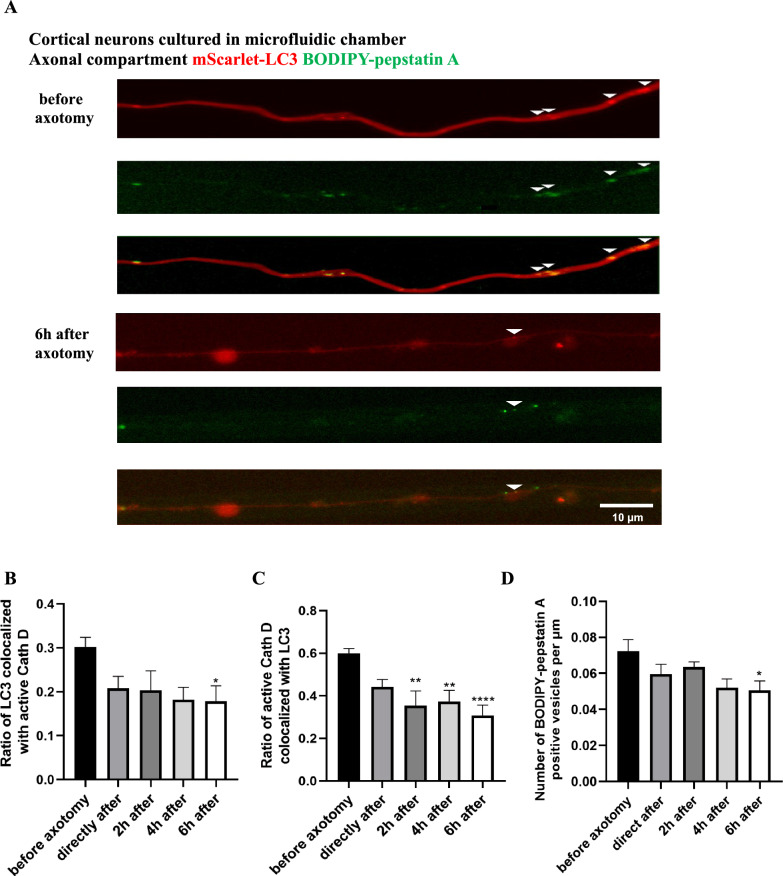
Colocalization analysis of LC3 and cathepsin D following axonal injury. **A** Representative images showing a partial colocalization of mScarlet-LC3 with active cathepsin D in the axons of cortical neurons before and 6 h after axotomy. Arrows indicate LC3-positive vesicles with detectable BODIPY–pepstatin A. **B** Quantitative analysis of mScarlet-LC3 labeled vesicles positive for active cathepsin D at the indicated time points. **C** Quantitative analysis of BODIPY–pepstatin A positive vesicles colocalized with LC3 labeled by the given viral vector at the indicated time points. **D** Quantification of the number of BODIPY-pepstain A labeled active cathepsin D at the given time points after axonal injury. Error bars represent Mean ± SEM. Data was quantified from at least 23 neuronal axons at each time point in three independent experiments. N.S. no significant difference; *P < 0.05; **P < 0.01; ***P < 0.001; ****P < 0.0001 by one-way ANOVA and Tukey multiple comparisons test or Kruskal–Wallis test and Dunn’s multiple comparisons test based on the normality test of variables

Cathepsin D is an aspartyl protease localized in lysosomes where it is activated upon intravesicular acidification to degrade the engulfed proteins. BODIPY–pepstatin A, tagged with a green fluorophore, binds specifically to active cathepsin D in acidified environments at the pH of 4.5 and can thus be used to detect acidified lysosomes. AAV.mScarlet-LC3 transduced primary cortical neurons in microfluidic chambers were incubated with BODIPY-pepstatin A in both soma and axonal compartments for 1 h. Then, imaging was conducted before and up to 6 h after axotomy on the axon side to trace the colocalization of mScarlet-LC3 and BODIPY-pepstatin A labeled active cathepsin D (Fig. 7A).

At basal conditions, the ratio of LC3 colocalized with active cathepsin D was 30 ± 2% of all labeled LC3 vesicles (Fig. 7B). On the other hand, 60 ± 2% of all active cathepsin D vesicles were also positive for LC3 (Fig. 7C). After axotomy, both numbers gradually dropped and reached their lowest point at 6 h after axotomy (18 ± 3% of all labeled LC3 vesicles colocalized with active cathepsin D, p = 0.0428; and 31 ± 5% of all active cathepsin D vesicles were also positive for LC3, p < 0.0001; Fig. 7B, C). Therefore, the fusion events of cathepsin D and LC3 positive vesicles were decreased after axotomy. Moreover, the absolute number of active cathepsin D positive vesicles was decreased up to 6 h after axotomy (Fig. 7D, p = 0.0285). No changes were detected in axons without axotomy over 6 h of imaging (Video S3 and Fig. S3).

To confirm these results, we transfected the cortical neurons with a mCherry-LC3-EGFP tandem construct that expresses both fluorophores together with LC3. Since EGFP is quenched at a pH < 5.0 whereas mCherry is not pH-sensitive, all LC3-positive AVs are labelled with mCherry but only the AV fraction excluding the more acidic autophagolysosomes also contain EGFP. We found a rapid significant decrease in the number of autophagolysosomes (i.e. mCherry-positive, EGFP negative vesicles as compared to all mCherry-positive vesicles) after axotomy which was persistent until 4 h after axotomy (Fig. S4).

These findings indicate that axotomy has a negative impact on the fusion of AVs and lysosomes containing mature hydrolases and on lysosome formation.

Interestingly, treatment of the neurons with the drug bafilomycin A1, which inhibits autophagosome-lysosome and V-ATPase-dependent acidification of AVs, led to similar changes in axonal AV transport as the axotomy (Fig. S5). This finding suggests that the blockage of AV-lysosome-fusion might be one of the main mechanisms underlying the effects of axotomy on axonal AV transport.

## Discussion

In this study, we characterized axonal AV transport in the rat optic nerve in vivo using live-imaging with a two-photon-microscope and viral vector mediated labeling of LC3-containing AVs. We were able to visualize intraaxonal moving and stationary AVs and quantify their transport parameters in the living animal. A crush lesion of the optic nerve resulted in a rapid breakdown of the retrograde axonal AV transport with only little effect on the anterograde transport. Although the formation of AVs in the lesioned axon was activated within several hours, their clearance was severely impaired. This was accompanied in vivo by a decrease of lysosomal markers in the perilesional axonal region and in vitro by a reduced acidification of the AVs and fusion with lysosomes following axotomy. Moreover, expression levels of the dynactin component p150Glued were significantly reduced after crush which could be a mechanistical link to the retrograde transport impairment.

To the best of our knowledge, this is the first study using live imaging to analyze axonal autophagic vesicle transport in the mammalian CNS in vivo. Compared to previous publications [20, 21] and our own in vitro data in primary cortical neurons presented here, the characteristics of axonal AV transport in the optic nerve in vivo were similar with some minor but significant differences. The overall density of AVs in the axons was comparable between in vivo and in vitro with a trend to a lower density in vivo, but this could depend on labeling efficacy. The amount of mobile AVs in vivo was also similar to in vitro with around half of the visible AVs being motile within a 5 min observation period in vivo and 65% in vitro. However, the predominance of the retrograde transport direction was more prominent in vivo than in vitro. The transport velocity of retrograde transport was significantly higher than of the anterograde transport in vivo, while there was no difference in vitro. Moreover, the effects of axotomy were much more pronounced in vivo than in vitro. We think that these differences reflect the fact that the optic nerve axons belong to differentiated neurons in a glial environment in the CNS of an adult animal, where transport characteristics have specifically evolved compared to primary embryonic neurons that are usually used for in vitro experiments. However, we cannot completely rule out that these differences are also at least partly caused by technical reasons including anesthesia of the animal and surgical manipulation of the optic nerve.

The primary cortical neurons employed here in vitro represent another neuronal cell type than the RGCs analyzed in vivo. Rat primary RGCs, the natural in vitro equivalent of the optic nerve axons in vivo, hardly grow axons through microgrooves which is essential for specific analysis of the axonal compartment, probably because their high sensitivity to the flow rate induced shear stress. Cortical neurons were hence chosen because they are a very well established and commonly used in vitro model. Also, they contain a variety of different neuronal cell types present in the cortex. Thus, our in vitro results should not be viewed as a pure in vitro equivalent for the in vivo experiments, but rather as an extension of the analysis to other neuronal cell types.

Mounting evidence suggests that autophagy plays a major role in axonal degeneration [22]. However, so far only a few studies have examined alterations in the kinetics of AV trafficking, most of which were obtained in vitro [20, 23]. Here, we find a significant decrease in the number of AVs undergoing retrograde transport, while anterogradely transported AVs and transport velocity remained stable. It was demonstrated before, that AVs are generated at the axon tip and are then transported towards the cell body [4, 24]. Thus, the blockage of axonal transport by the lesion and yet not so rapidly initiated AVs biogenesis at the tip or the axonal stump may be reasons for reduced retrograde transport. On the other hand, we could demonstrate that AV formation at the proximal axonal end is resumed and activated within 6 h after ONC in vivo, as indicated by the increased p-Atg16L1 expression [16, 25] and the increased LC3-II/LC3-I ratio. Additionally, there is a sustained increase in stationary AVs after ONC. We therefore think that impaired retrograde axonal transport in the proximal axon is the main reason for the observed effects while AV biogenesis is resumed rapidly.

The mechanisms underlying the retrograde transport deficit are not yet completely understood. One reason might be a local energy deficit that is known to occur after axonal injury due to mitochondrial damage, decreased mitochondrial transport, and increased energy consumption [26]. Unlike the unidirectional stepping of kinesin-1 motors, recent in vitro and in vivo measurements indicate that retrograde transport manifests itself as movement in teams of 6–12 motors, which is not only less efficient than anterograde transport but also requires more energy expenditure [5]. In this context, retrograde transport is more likely to be impaired than anterograde transport in the context of energy deficiency after axonal injury.

Considering that AV-lysosome fusion is a prerequisite for the degradation of autophagic substrates and the clearance of autophagic vesicles, the expression of the lysosomal protein cathepsin D and the fusion ratio were evaluated. Although LAMP1 is often used as a lysosomal marker, a significant proportion of LAMP1-labeled vesicles do not contain lysosomal hydrolases, implying that they are actually not lysosomes [27]. To this end, cathepsin D, an aspartyl protease that primarily resides within lysosomes, was used to more specifically label lysosomes in this study. After axotomy, we observed a significantly reduced fusion of AVs and lysosomes in primary cortical neurons in vitro and reduced cathepsin expression levels in the perilesional axonal region in vivo. This suggests that that axonal injury impairs the maturation of AVs, leading to a decrease in mature autolysosomes. Since efficient autophagic degradation requires the fusion of AVs with multiple late endosomes/lysosomes to maintain an adequately acidic environment for hydrolases to function [28, 29], AVs are unable to turn over and accumulate as nondegradable autophagic vacuoles, which have been widely reported in both neurodegenerative diseases and traumatic neuropathies [30–32].

Protein levels of cathepsin D were evaluated following axonal injury. At 6 h after the crush lesion, native cathepsin D levels were exclusively reduced on the distal side. The last finding could be explained by an ongoing supplementation of the proximal axon with native cathepsin that is interrupted on the distal side. On both sides of the crush, mature cathepsin D levels were significantly decreased which suggests that acidification of lysosomes and formation of autophagolysosomes are impaired after axotomy. Decreased levels and activity of the lysosomal protein cathepsin D were also recently reported in CNS trauma, such as traumatic brain injury and spinal cord injury, frequently accompanied by inhibition of autophagy flux [33, 34]. However, since intraluminal acidification [35] and microtubule-dependent axonal transport [36, 37] are both essential for the successful function of the lysosomal digestive system, it still remains to be clarified by further research which process is dominant or whether this is a result of their interaction.

Furthermore, since crush lesion selectively disrupted the retrograde axonal transport in the optic nerve, we tried to explore the underlying molecular mechanisms. After assessing the levels of several key motor proteins involved in AV transport, a significant reduction in p150Glued was found after axonal injury, but no significant difference in dynein. P150Glued is the largest subunit of the dynactin complex, which facilitates dynein activation and, together with dynein, effectively enables the long-distance retrograde transport of cargo [38, 39]. Hence, p150Glued deficiency could reasonably explain our previous finding of severely impaired retrograde transport following crush lesion in the optic nerve. Consistently, recently published studies have also shown that the G59S mutation in p150Glued and the depletion of p150Glued leads to a partial loss of capacity of dynein/dynactin-mediated retrograde transport and the accumulation of retrogradely transported cargoes, such as synaptophysin or autophagosomes, thus exacerbating neurodegeneration [40–42]. To date, it is unknown how the ONC causes the reduction in p150Glued. In fact, compared to dynein, dynactin1/p150Glued is more vulnerable to toxic exposures, especially excitotoxicity [43, 44]. Recently, a growing body of evidence has suggested that glutamate levels appear elevated after traumatic ONC, including upregulation of glutamate transporter mRNA [45, 46]. Moreover, the administration of glutamate antagonists or blockers of glutamate receptors provided neuroprotective effects [47, 48]. Accordingly, we hypothesize that glutamate excitotoxicity induced by optic nerve injury may adversely affect p150Glued. Interestingly, Fujiwara et al. found that glutamate excitotoxicity generated a C-terminal truncated form of p150Glued, thus exacerbating axonal degeneration. Consistently, overexpression of p150Glued attenuated axonal degeneration and suppressed cell body death [49, 50]. However, the study by Fujiwara et al. also revealed a 35% drop in the dynein intermediate chain (DIC), which is different from our results. Therefore, lesion-induced glutamate excitotoxicity alone may not adequately explain our findings. Another possible explanation is that p150Glued is cleaved by proteases or caspases as demonstrated before [51]. The detailed molecular mechanisms underlying the retrograde transport impairment after ONC still need to be elucidated.

Based on our results, an enhancement of p150Glued could be a therapeutic approach to attenuate axonal transport impairment and axonal degeneration after lesion. Accordingly, preliminary experiments from our lab indicate that overexpression of p150Glued can rescue axonal transport deficits after axotomy, but this needs to be confirmed in future experiments.

In summary, we demonstrate here a more differentiated axonal transport of AVs in the rat optic nerve in vivo as compared to in vitro. After axotomy, retrograde but not anterograde transport of AVs is severely impaired although formation of autophagosomes is activated. The retrograde transport impairment is associated with a decrease of p150Glued, an adaptor protein of the major protein dynein. Moreover, lysosomal markers are reduced implying a defective formation of autophagolysosomes and clearance of autophagosomes.

### Supplementary Information

Fig. S1 Trafficking of autophagic vesicles in the rat optic nerve over 6 h *in vivo* without a lesion (A, B, C) Quantification of the number of motile and stationary LC3 vesicles in the optic nerve of rats at the given time points. Error bars represent Mean ± SEM. (D) Quantification of the average velocity of different moving LC3 vesicles at the given time points. In all quantifications, a minimum of 10 axons per time point per animal was evaluated and a total of 3 animals were included. Significance was determined by paired test or Wilcoxon test based on the normality test of variables. No significant differences were detected at any time-point or in any parameter, suggesting that two-photon imaging up to 6 hours after optic nerve exposure did not affect autophagosome transport. (PDF 148 kb).

Fig. S2 Immunofluorescence staining of p150Glued, Cathepsin D and STX17 in different regions of the rat optic nerve following crush lesion. (A) Representative immunofluorescence staining of p150glued (green), Cathepsin D (green), STX 17 (red; axons labeled by intravitreal injected EGFP virus in green), and DAPI (blue) in the contralateral unlesioned control and different parts of the optic nerve 6 hours after crush injury. The lower right corner of the image showed the enlarged area in the corresponding dotted box. Scale bar: 5 μm. (B, C, D) Quantitative analysis of the number of p150Glued, Cathepsin D and axonal STX17 puncta in different regions (proximal and distal to 500 μm away from the crush site) compared to contralateral uninjured control Error bars represent Mean ± SEM. Data were quantified from 3 animals, with 5–7 views evaluated for each region in individual animal. N.S. no significant difference; **P < 0.01; ***P < 0.001; ****P < 0.0001 by one-way ANOVA and Tukey multiple comparisons test or Kruskal–Wallis test and Dunn’s multiple comparisons test based on the normality test of variables. (PDF 805 kb).

Fig. S3 Colocalization of LC3 and active Cath D in cortical neurons over 6-hour imaging. (A, B) Quantification of the number of LC3 and active cathepsin D vesicles per μm in cortical neuronal axons at the given time points. (C) Quantitative analysis of mScarlet-LC3 labeled vesicles positive for active cathepsin D at the indicated time points. (D) Quantitative analysis of BODIPY–pepstatin A positive vesicles colocalized with LC3 labeled by the given viral vector at the indicated time points. Error bars represent mean ± SEM. No statistical significance was found by one-way ANOVA and Tukey multiple comparisons test or Kruskal–Wallis test and Dunn’s multiple comparisons test based on the normality test of variables. (PDF 134 kb).

Fig. S4 Fusion of autophagic vesicles with lysosomes in primary cortical neurons before and after axotomy. (A) Representative kymographs of mCherry-GFP-LC3-labeled autophagic vesicles along axons before (left panel) and 4 h after axotomy (right panel). Scale bar: 10 μm. (B) Quantification of th ratio of mCherry-only positive vesicles to mCherry LC3-positive vesicles. Error bars represent mean ± SEM. Data was quantified from at least 15 neuronal axons at each time point in three independent experiments.. All statistical analyses were performed by comparing the data at different time points after axotomy to the data before axotomy. N.S. no significant difference; *P < 0.05; **P < 0.01; by one-way ANOVA and Tukey multiple comparisons test or Kruskal–Wallis test and Dunn’s multiple comparisons test based on the normality test of variables. (PDF 275 kb).

Fig. S5 Live imaging of autophagosomes in rat primary cortical neurons after bafilomycin A1 treatment. A) Exemplary kymographs of autophagosomes along the axons before and after 30 h incubation with bafilomycin A1 (5 nm) treatment (x-axis: length of the axon; y-axis: time). (B, C, D) Quantification of autophagosome movements before and after bafilomycin A1 treatment. Error bars represent mean ± SEM. (E) Quantification of the average velocity of autophagosomes before and after bafilomycin treatment. Data was quantified from at least 27 neuronal axons at each time point in three independent experiments. **P < 0.01; ****P < 0.0001 by unpaired t test or Mann–Whitney test according to the normality tests. (PDF 237 kb).

**Video S1** Representative two-photon live-imaging of the optic nerve before and 6 h after crush lesion in young rats. The somatic side is toward the left. **Video S2** Representative two-photon live-imaging of the optic nerve over 6 hours without crush lesion. The somatic side is toward the left. **Video S3** Fusion of mScarlet-LC3 (red) and active cathepsin D visualized by BODIPY-pepstatin A (green) in microfluidic chambers. The somatic side is toward the left. (PPTX 69915 kb).

## Data Availability

All data that are not already available as part of the publication or the supplements are provided upon request by the corresponding author.

## Abbreviations

AAD: Acute axonal degeneration
AAV: Adeno-associated virus
AVs: Autophagic vesicles
CNS: Central nervous system
DIV: Day in vitro
ONC: Optic nerve crush
RGC: Retinal ganglion cells
