# Supplementary material for: Axonal autophagic vesicle transport in the rat optic nerve in vivo under normal conditions and during acute axonal degeneration
Source: Acta Neuropathol Commun. 2024 May 29;12:82. doi: 10.1186/s40478-024-01791-2 (PMC11134632; doi:10.1186/s40478-024-01791-2)
Supplement: Supplementary file 1 — Trafficking of autophagic vesicles in the rat optic nerve over 6 h in vivo without a lesion (A, B, C) Quantification of the number of motile and stationary LC3 vesicles in the optic nerve of rats at the given time points. Error bars represent Mean ± SEM. (D) Quantification of the average velocity of different moving LC3 vesicles at the given time points. In all quantifications, a minimum of 10 axons per time point per animal was evaluated and a total of 3 animals were included. Significance was determined by paired test or Wilcoxon test based on the normality test of variables. No significant differences were detected at any time-point or in any parameter, suggesting that two-photon imaging up to 6 hours after optic nerve exposure did not affect autophagosome transport. (PDF 148 kb). [file 40478_2024_1791_MOESM1_ESM.pdf]

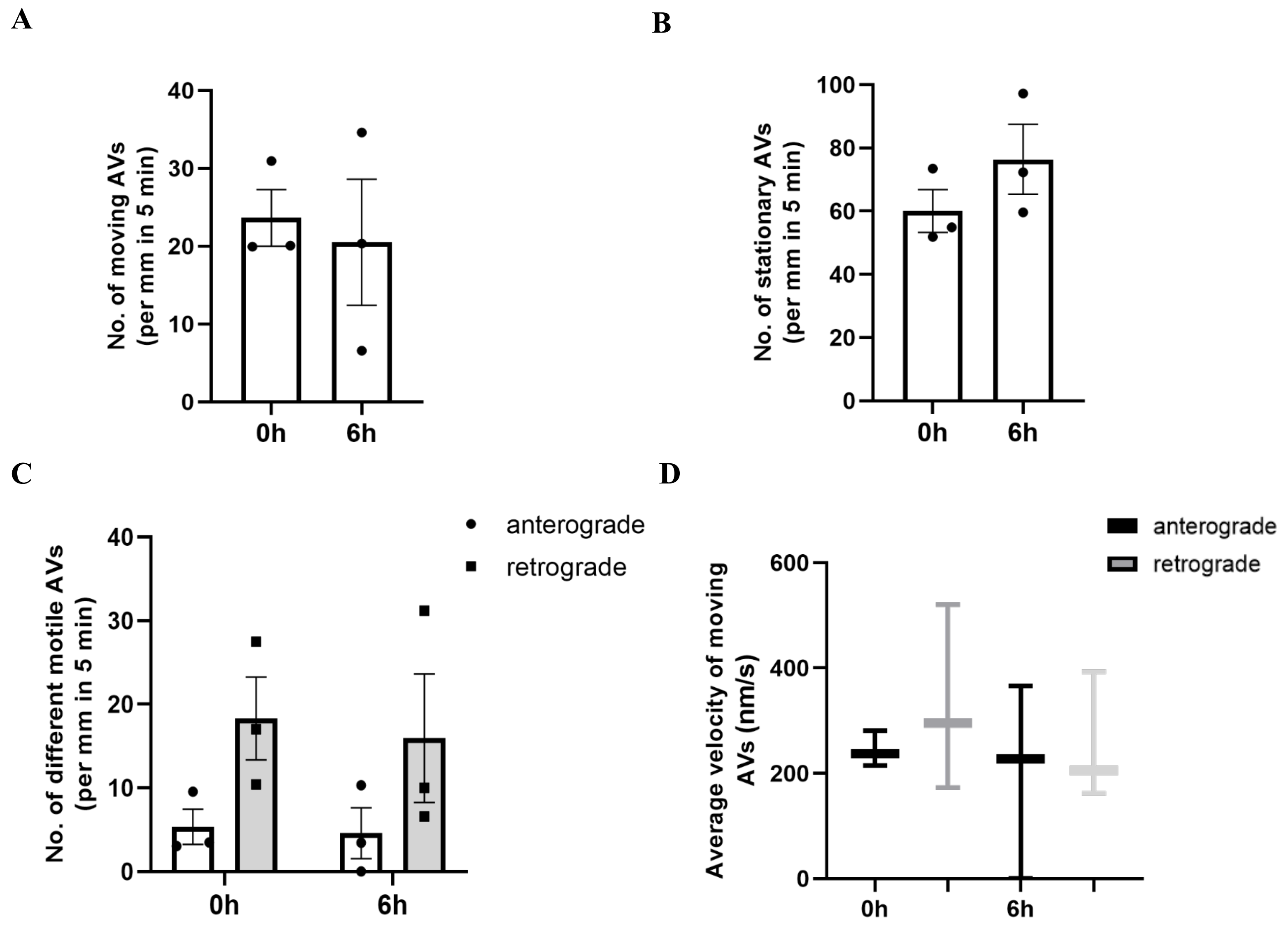

A
No. of moving AVs (per mm in 5 min)
0
10
20
30
40
0h
6h
B
No. of stationary AVs (per mm in 5 min)
0
20
40
60
80
100
0h
6h
C
No. of different motile AVs (per mm in 5 min)
anterograde
retrograde
0
10
20
30
40
0h
6h
D
Average velocity of moving AVs (nm/s)
anterograde
retrograde
0
200
400
600
0h
6h
