# Supplementary figures and images for: Axonal autophagic vesicle transport in the rat optic nerve in vivo under normal conditions and during acute axonal degeneration
Source: Acta Neuropathol Commun. 2024 May 29;12:82. doi: 10.1186/s40478-024-01791-2 (PMC11134632; doi:10.1186/s40478-024-01791-2)

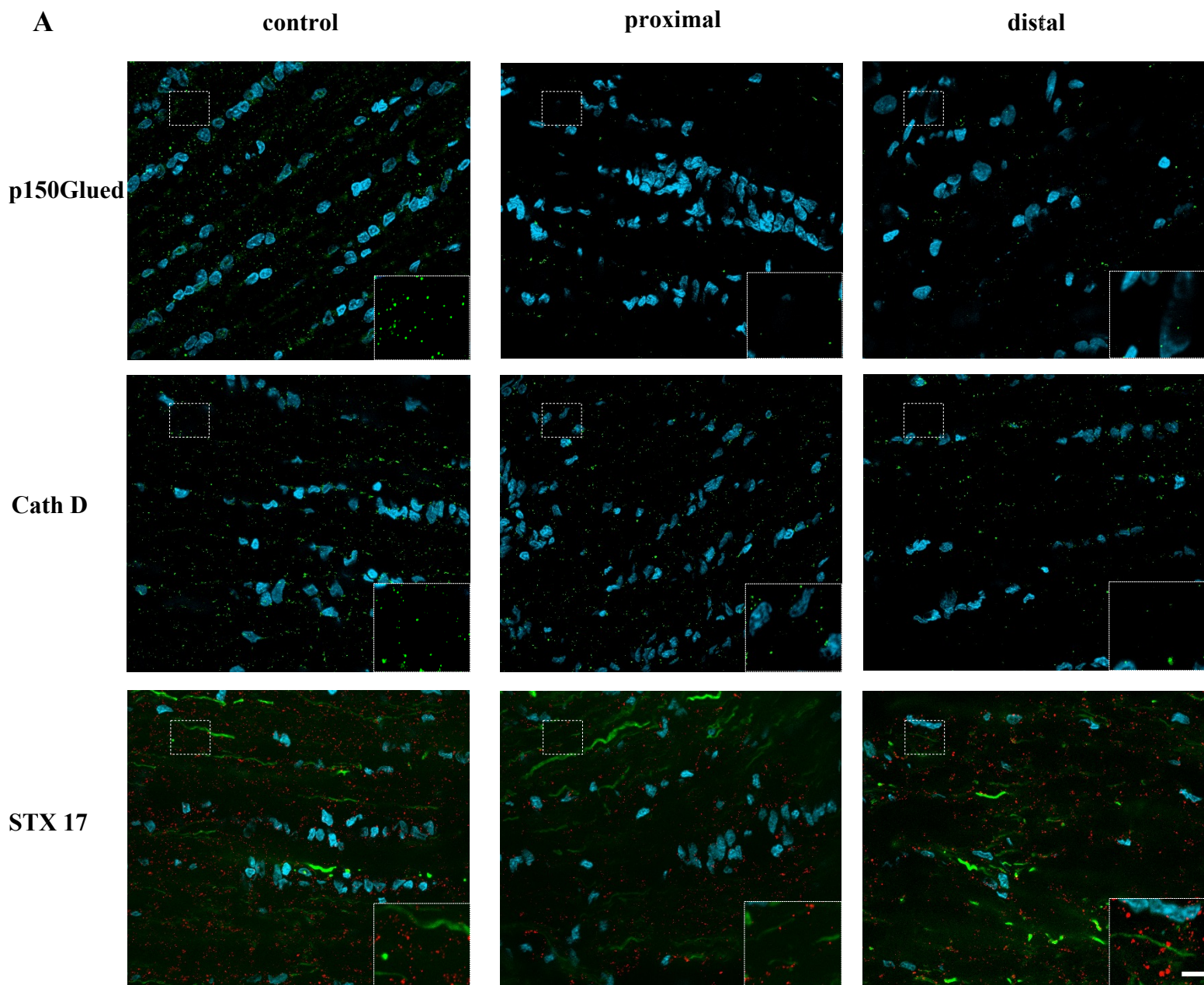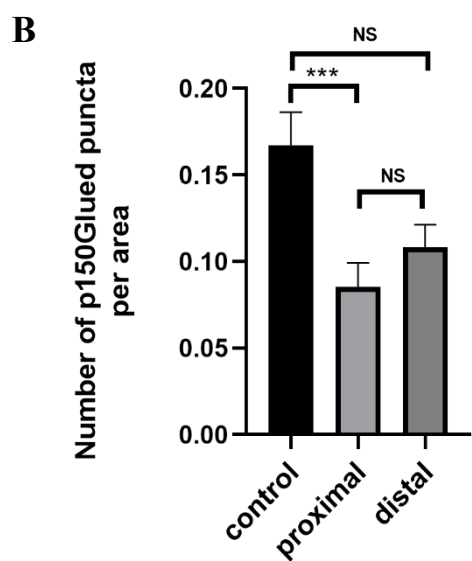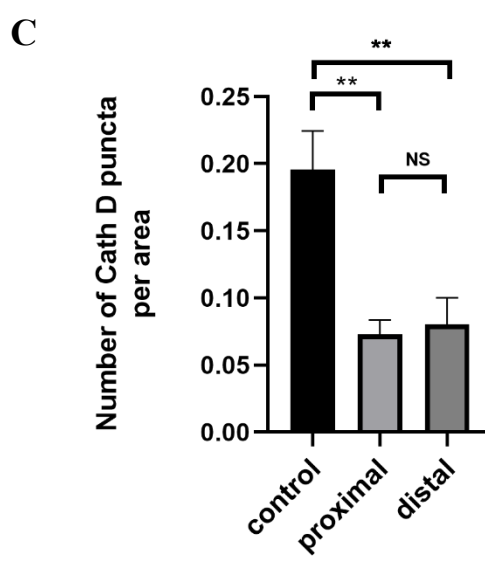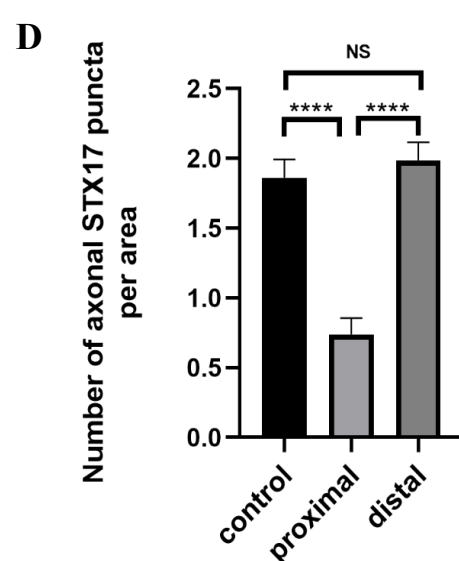

Supplement: Supplementary file 2 — Immunofluorescence staining of p150Glued, Cathepsin D and STX17 in different regions of the rat optic nerve following crush lesion. (A) Representative immunofluorescence staining of p150glued (green), Cathepsin D (green), STX 17 (red; axons labeled by intravitreal injected EGFP virus in green), and DAPI (blue) in the contralateral unlesioned control and different parts of the optic nerve 6 hours after crush injury. The lower right corner of the image showed the enlarged area in the corresponding dotted box. Scale bar: 5 μm. (B, C, D) Quantitative analysis of the number of p150Glued, Cathepsin D and axonal STX17 puncta in different regions (proximal and distal to 500 μm away from the crush site) compared to contralateral uninjured control Error bars represent Mean ± SEM. Data were quantified from 3 animals, with 5–7 views evaluated for each region in individual animal. N.S. no significant difference; **P < 0.01; ***P < 0.001; ****P < 0.0001 by one-way ANOVA and Tukey multiple comparisons test or Kruskal–Wallis test and Dunn’s multiple comparisons test based on the normality test of variables. (PDF 805 kb). [file 40478_2024_1791_MOESM2_ESM.pdf]

**A**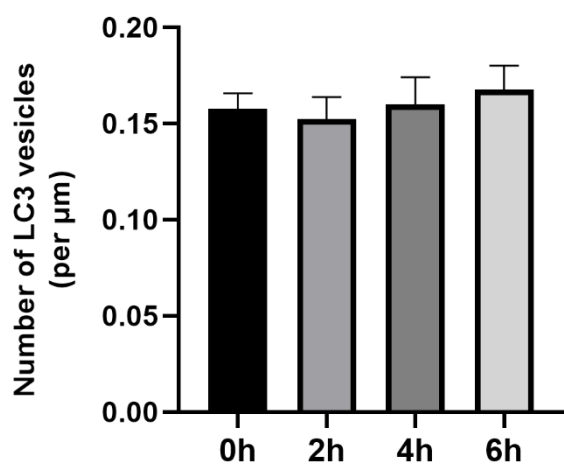**B**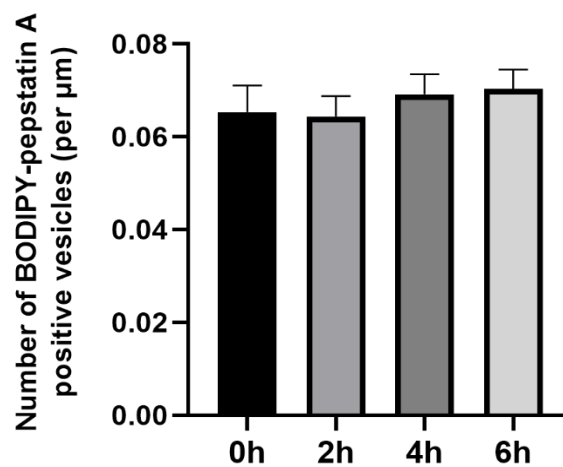**C**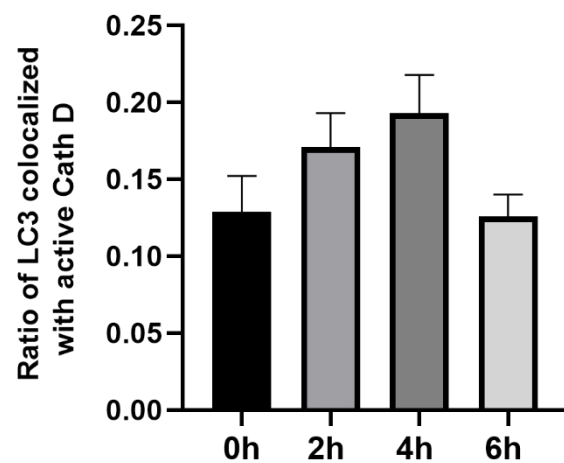**D**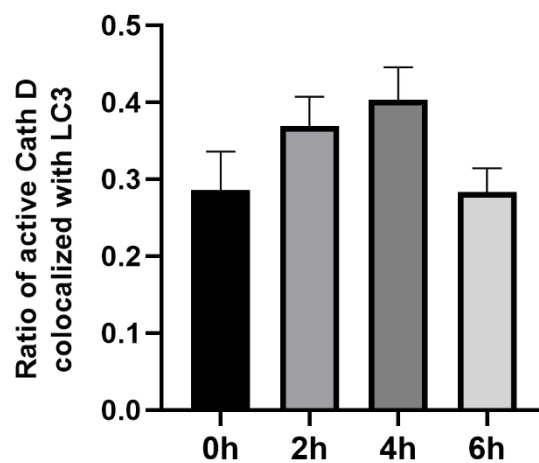

Supplement: Supplementary file 3 — Colocalization of LC3 and active Cath D in cortical neurons over 6-hour imaging. (A, B) Quantification of the number of LC3 and active cathepsin D vesicles per μm in cortical neuronal axons at the given time points. (C) Quantitative analysis of mScarlet-LC3 labeled vesicles positive for active cathepsin D at the indicated time points. (D) Quantitative analysis of BODIPY–pepstatin A positive vesicles colocalized with LC3 labeled by the given viral vector at the indicated time points. Error bars represent mean ± SEM. No statistical significance was found by one-way ANOVA and Tukey multiple comparisons test or Kruskal–Wallis test and Dunn’s multiple comparisons test based on the normality test of variables. (PDF 134 kb). [file 40478_2024_1791_MOESM3_ESM.pdf]

**A**

control

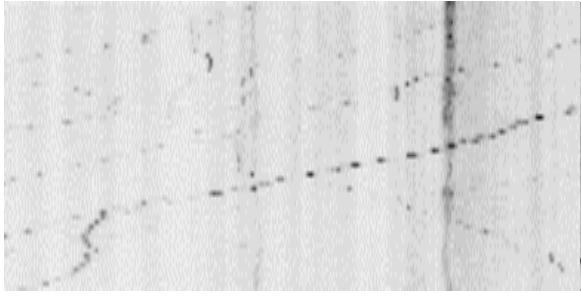

bafilomycin

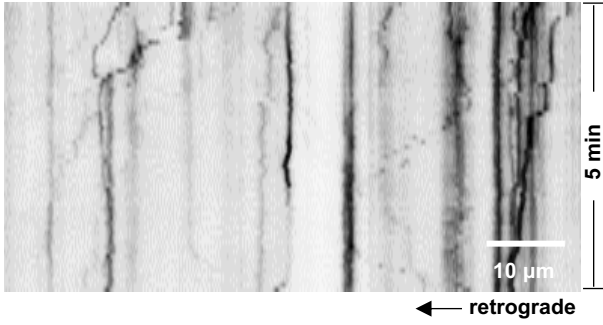

**B**

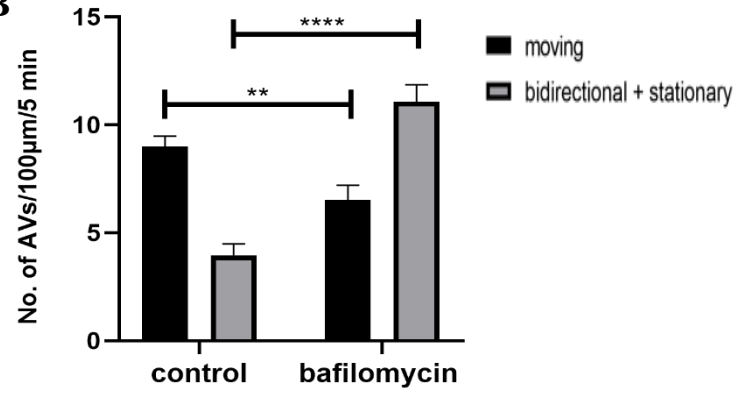

**C**

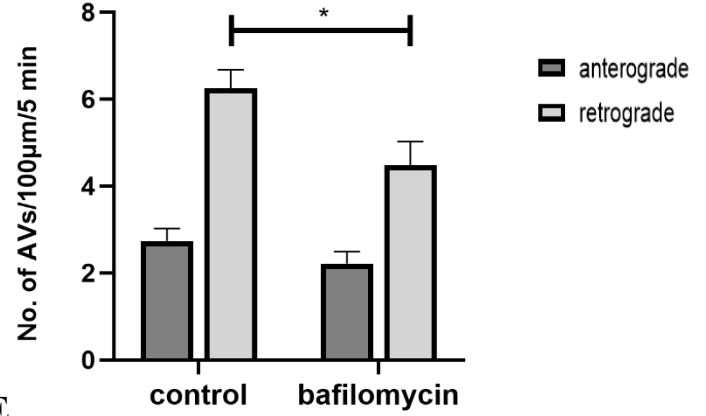

**D**

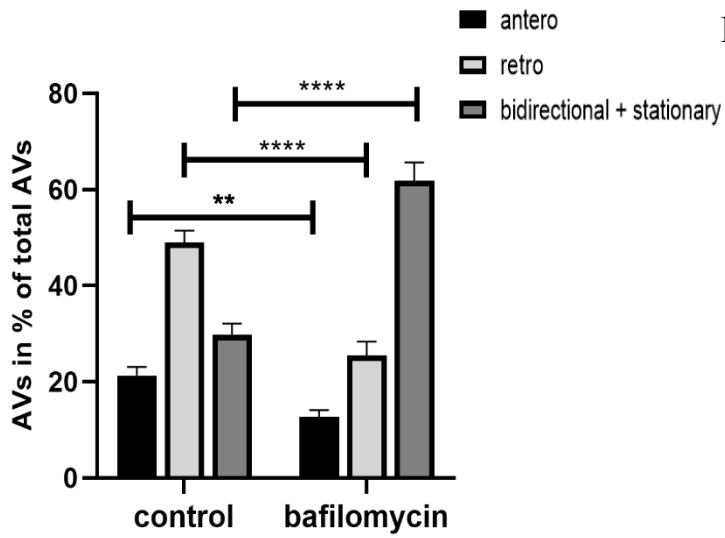

**E**

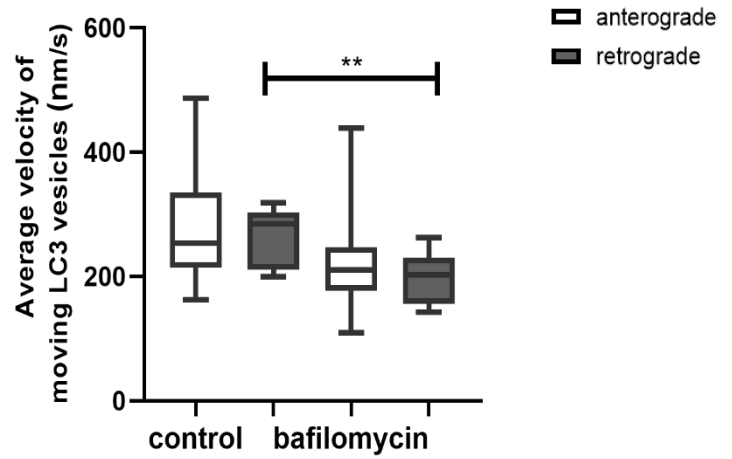

Supplement: Supplementary file 5 — Live imaging of autophagosomes in rat primary cortical neurons after bafilomycin A1 treatment. A) Exemplary kymographs of autophagosomes along the axons before and after 30 h incubation with bafilomycin A1 (5 nm) treatment (x-axis: length of the axon; y-axis: time). (B, C, D) Quantification of autophagosome movements before and after bafilomycin A1 treatment. Error bars represent mean ± SEM. (E) Quantification of the average velocity of autophagosomes before and after bafilomycin treatment. Data was quantified from at least 27 neuronal axons at each time point in three independent experiments. **P < 0.01; ****P < 0.0001 by unpaired t test or Mann–Whitney test according to the normality tests. (PDF 237 kb). [file 40478_2024_1791_MOESM5_ESM.pdf]

## Slide 1
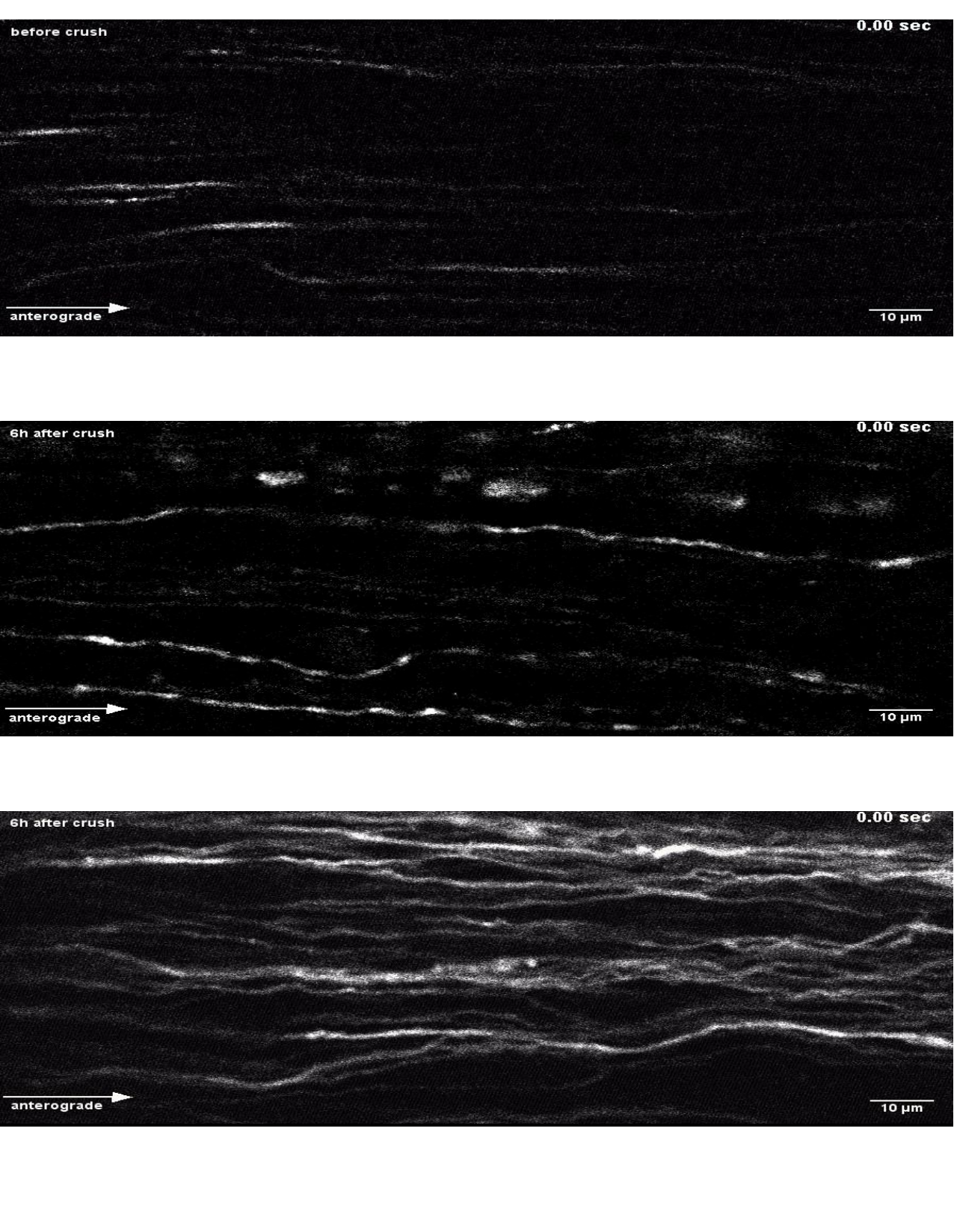

10 μm

## Slide 2
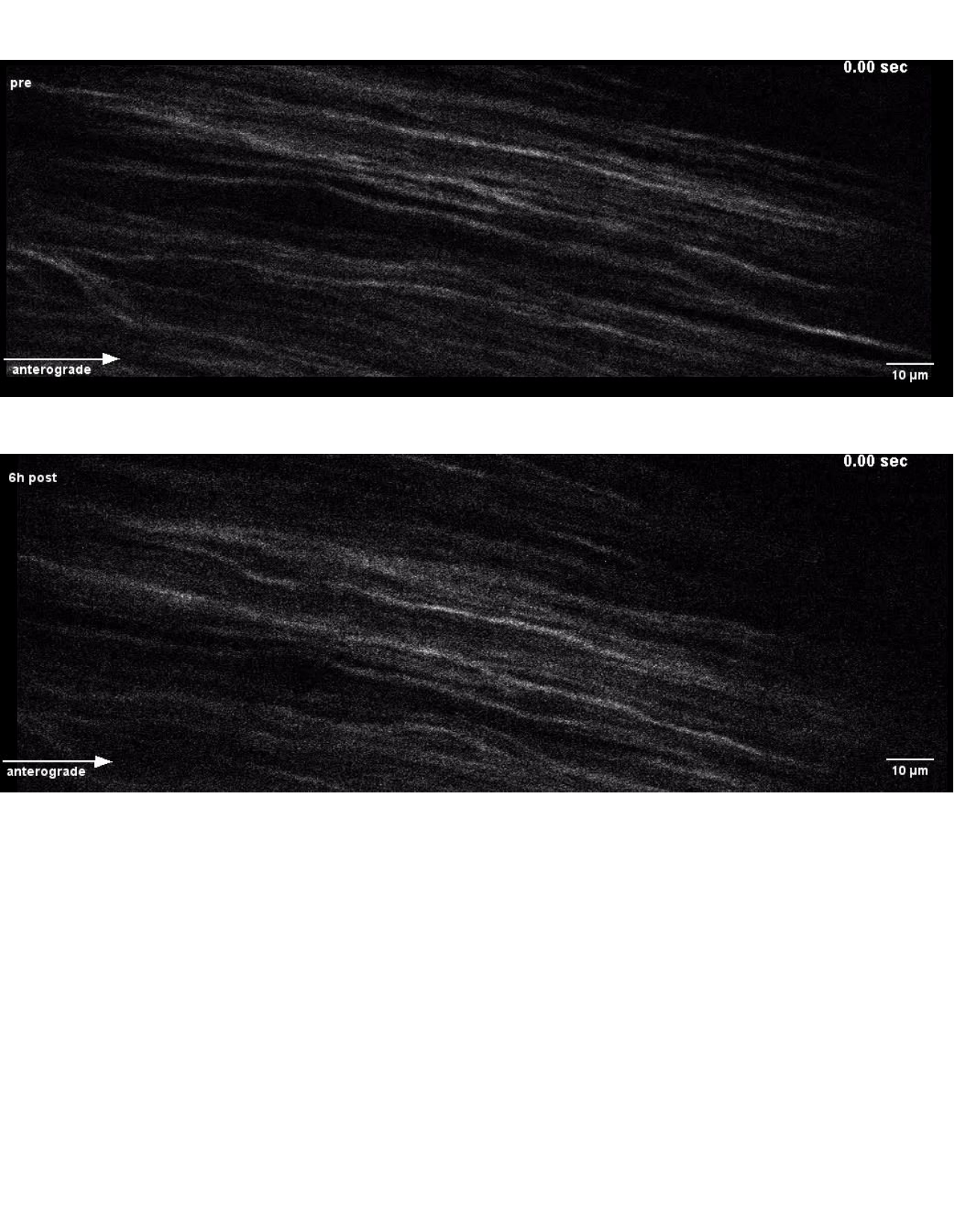

10 μm
10 μm

## Slide 3
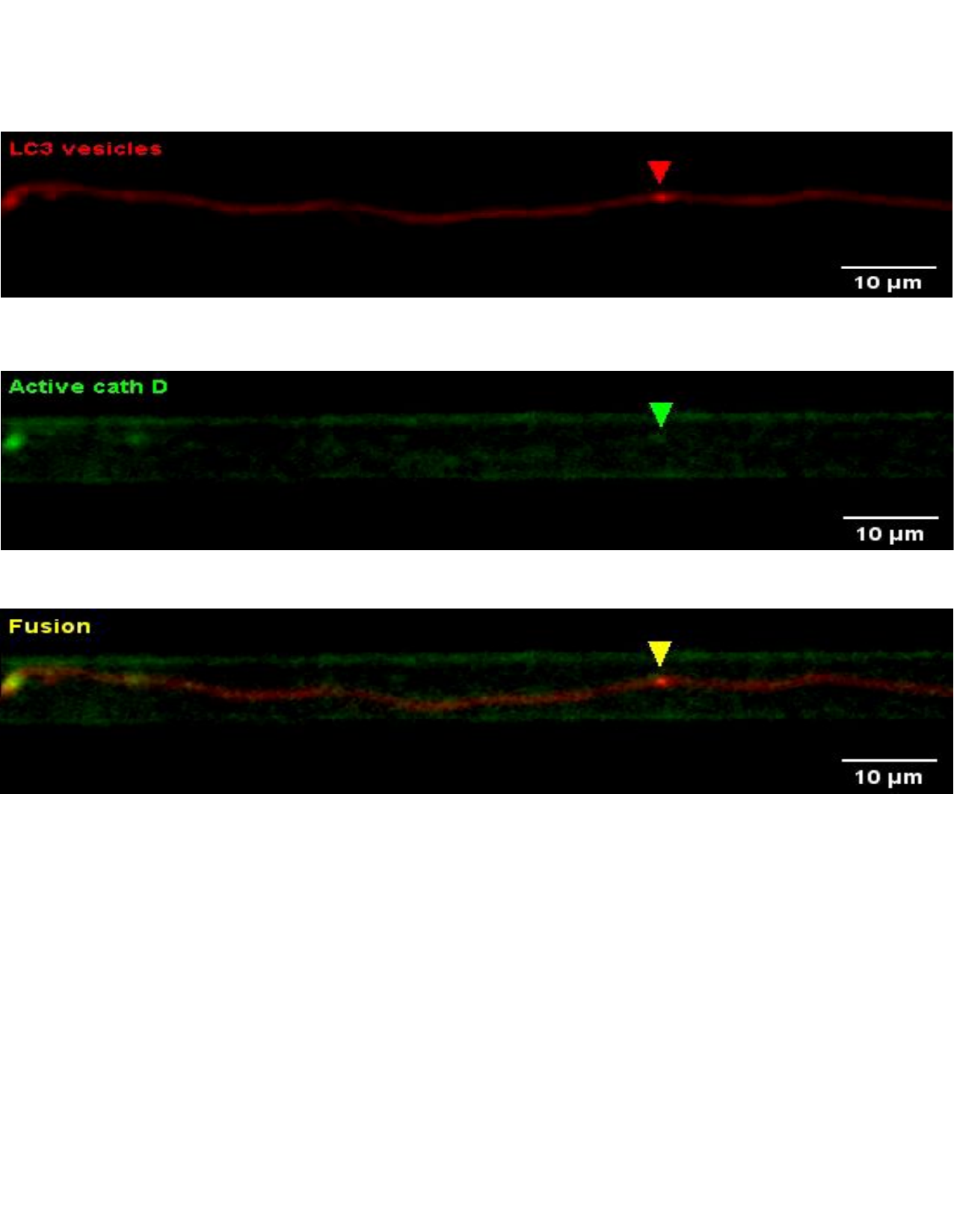

10 μm
10 μm

Supplement: Supplementary file 6 — Video S1 Representative two-photon live-imaging of the optic nerve before and 6 h after crush lesion in young rats. The somatic side is toward the left. Video S2 Representative two-photon live-imaging of the optic nerve over 6 hours without crush lesion. The somatic side is toward the left. Video S3 Fusion of mScarlet-LC3 (red) and active cathepsin D visualized by BODIPY-pepstatin A (green) in microfluidic chambers. The somatic side is toward the left. (PPTX 69915 kb). [file 40478_2024_1791_MOESM6_ESM.pptx]
