# Supplementary material for: Axonal autophagic vesicle transport in the rat optic nerve in vivo under normal conditions and during acute axonal degeneration
Source: Acta Neuropathol Commun. 2024 May 29;12:82. doi: 10.1186/s40478-024-01791-2 (PMC11134632; doi:10.1186/s40478-024-01791-2)
Supplement: Supplementary file 4 — Fusion of autophagic vesicles with lysosomes in primary cortical neurons before and after axotomy. (A) Representative kymographs of mCherry-GFP-LC3-labeled autophagic vesicles along axons before (left panel) and 4 h after axotomy (right panel). Scale bar: 10 μm. (B) Quantification of th ratio of mCherry-only positive vesicles to mCherry LC3-positive vesicles. Error bars represent mean ± SEM. Data was quantified from at least 15 neuronal axons at each time point in three independent experiments.. All statistical analyses were performed by comparing the data at different time points after axotomy to the data before axotomy. N.S. no significant difference; *P < 0.05; **P < 0.01; by one-way ANOVA and Tukey multiple comparisons test or Kruskal–Wallis test and Dunn’s multiple comparisons test based on the normality test of variables. (PDF 275 kb). [file 40478_2024_1791_MOESM4_ESM.pdf]

A

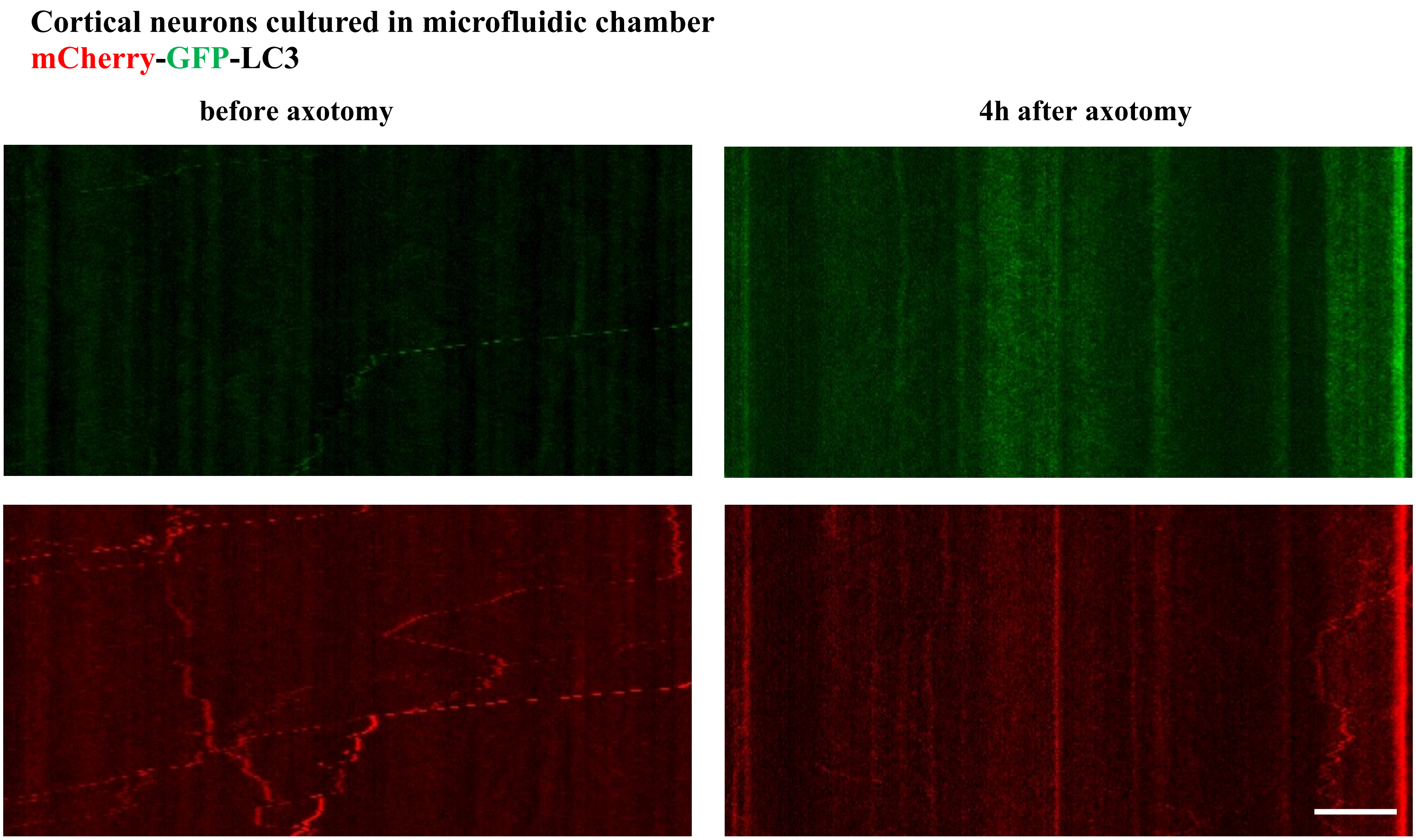
Cortical neurons cultured in microfluidic chamber
mCherry-GFP-LC3
before axotomy
4h after axotomy

B

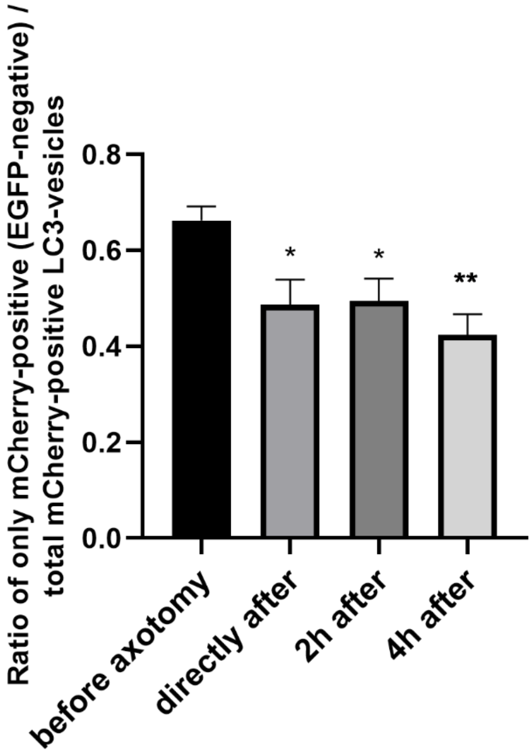
Ratio of only mCherry-positive (EGFP-negative) / total mCherry-positive LC3-vesicles
0.8
0.6
0.4
0.2
0.0
*
*
**
before axotomy
directly after
2h after
4h after
